# Multiple co-clustering based on nonparametric mixture models with heterogeneous marginal distributions

**DOI:** 10.1371/journal.pone.0186566

**Published:** 2017-10-19

**Authors:** Tomoki Tokuda, Junichiro Yoshimoto, Yu Shimizu, Go Okada, Masahiro Takamura, Yasumasa Okamoto, Shigeto Yamawaki, Kenji Doya

**Affiliations:** 1 Okinawa Institute of Science and Technology Graduate University, 1919-1, Tancha, Onna-son, Okinawa, 904-0495, Japan; 2 Nara Institute of Science and Technology, 8916-5, Takayama-cho, Ikoma, Nara, 630-0192, Japan; 3 Department of Psychiatry and Neurosciences, Hiroshima University, Kasumi 1-2-3, Minami-ku, Hiroshima, 734-8551, Hiroshima, Japan; Jiangnan University, CHINA

## Abstract

We propose a novel method for multiple clustering, which is useful for analysis of high-dimensional data containing heterogeneous types of features. Our method is based on nonparametric Bayesian mixture models in which features are automatically partitioned (into views) for each clustering solution. This feature partition works as feature selection for a particular clustering solution, which screens out irrelevant features. To make our method applicable to high-dimensional data, a co-clustering structure is newly introduced for each view. Further, the outstanding novelty of our method is that we simultaneously model different distribution families, such as Gaussian, Poisson, and multinomial distributions in each cluster block, which widens areas of application to real data. We apply the proposed method to synthetic and real data, and show that our method outperforms other multiple clustering methods both in recovering true cluster structures and in computation time. Finally, we apply our method to a depression dataset with no true cluster structure available, from which useful inferences are drawn about possible clustering structures of the data.

## Introduction

We consider a clustering problem for a data matrix that consists of objects in rows and features (variables, or attributes) in columns. Clustering objects based on the data matrix is a basic data mining approach, which groups objects with similar patterns of distribution. As an extension of conventional clustering, a co-clustering model has been proposed which captures not only object cluster structure, but also feature cluster structure [1–3]. A survey paper by [4] provides a comprehensive picture of the concept of co-clustering. In principle, several types of co-clustering structure can be considered in terms of the way how a particular matrix entry is relevant for co-clustering structure: relevant only for a single co-cluster; relevant for more than one co-cluster (overlapping); not relevant for any co-cluster. As regards algorithms for inferring co-clustering structure, several approaches have been proposed, which can be categorized into model-based (assuming particular probabilistic distributions in each co-cluster) and non model-based (not explicitly assuming probabilistic distribution). Those algorithms include methods based on correlation coefficient [5] and factor analysis [6, 7].

**Fig 1:**
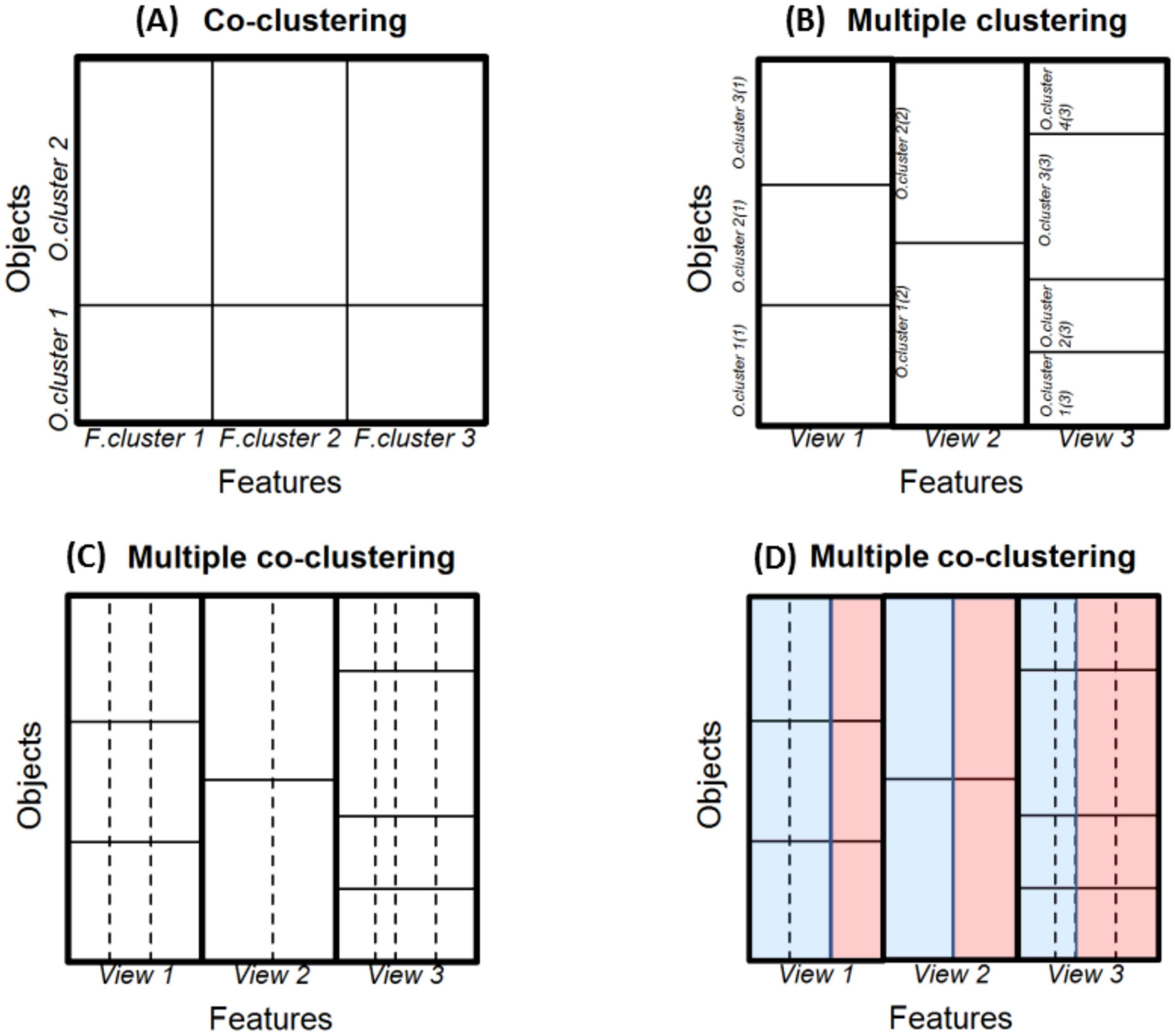
Illustration of clustering structures. Panel (A) co-clustering; (B) multiple clustering (with full covariance of Gaussian); (C) multiple clustering with a specific structure of co-clustering; (D) extension of the model (C) where different distribution families are mixed (two distributions families in blue and red). Note that a rectangle surrounded by bold lines corresponds to a single co-clustering structure with a single object cluster solution. In these panels, features and objects are sorted in the order of view, feature and object cluster indices (hence, the order of objects differs among the co-clustering rectangles).

In the present paper, we focus on a specific type of co-clustering, so called ‘check board’ [4] where both objects and features are exclusively partitioned (features are partitioned based on their distribution patterns, Fig 1A). This has an effect of reducing the number of parameters, which enables the model to fit high-dimensional data. Yet, the co-clustering method (as well as conventional clustering methods) does not always work well for real data, because real data may have different ‘views’ that characterize multiple clustering solutions (Fig 1B; here we use a terminology of ‘clustering’, meaning the whole set of clusters in a view) [8, 9].

To detect multiple clustering solutions of objects, several methods have recently been proposed [10]. Note that our purpose here is not to combine multiple views to generate a single clustering solution [11, 12], but to find multiple clustering solutions without prior knowledge of view structure. Relevant methods are mainly characterized by a guiding principle that underlies relationships among multiple clustering solutions (Table 1).

**Table 1:** Type of multiple clustering methods.

Principle	Description	Method
Dissimilarity	Given a clustering solution, another clustering solution should be dissimilar.	COALA [13]
		Constrained optimization [14]
		MAXIMUS [15]
Decomposition	Decompose a generative model into independent sub-models that yields each clustering solution.	Decorrelated *K*-means [16]
		Convolutional EM [16]
		CAMI [17]
Orthogonality	Identify orthogonal subspace for clustering solutions.	Orthogonal view approach [18]
		Multivariate Gaussian mixture [19]

A first batch of methods is based on dissimilarly between clustering solutions. In this group of methods, we first obtain a cluster solution by an arbitrary clustering method, followed by identifying a dissimilar clustering solution in a specific manner. COALA [13] aims to find a dissimilar clustering solution by imposing the constraint that a pair of objects should not belong to the same cluster in different clustering solutions (using a hierarchical clustering method). In the same sprit, constrained optimization method by [14] uncovers another clustering solution by transforming a data matrix while keeping balance between preservation of the original data structure and elimination of the given cluster structure. Further, MAXIMUS algorithm [15] identifies a dissimilar clustering solution based on spatial characteristics of a given clustering solution and a targeted clustering solution.

A second batch of methods decomposes a generative model of data into independent sub-models, aiming to simultaneously identify multiple clustering solutions. Decorrelated *K*-means algorithm [16] aims to find multiple clustering solutions based on *K*-means algorithm to minimize correlations among centroids. In the same sprit, convolutional EM algorithm [16] identifies multiple clustering solutions by modeling a generative distribution as sum of independent mixture models. CAMI [17] algorithm approaches this problem based on a probabilistic model to maximize log-likelihood of clustering solutions and minimize mutual information between them.

A third batch of methods considers orthogonal subspace of features for clustering. Orthogonal view approach by [18] performs an iterative algorithm for this purpose. Given a clustering solution, a next cluster solution is identified in orthogonal subspace of the current clustering solution. Simultaneous version of this type of method is proposed by [19], which is based on multivariate Gaussian mixture models (we discuss this method more in detail later).

Note that in this literature review, we did not include subspace clustering methods [20], because subspace clustering differs from multiple view clustering in that each cluster is embedded in different subspace. However, our interest in the present study is to find multiple clustering solutions in which each clustering solution identifies clusters embedded in the same subspace of features. We further clarify differences between our approach and subspace clustering in section of Simulation study on synthetic data.

For most of these multiple clustering methods, however, it is not straightforward to determine the number of views. A more promising approach is based on nonparametric mixture models assuming multivariate Gaussian mixture models for each view (Fig 1B) [19]. In this approach, the full Gaussian model for covariance matrices is considered, and the numbers of views and of object clusters are inferred in a data-driven way via the Dirichlet process. Such a method is quite useful to discover possible multiple cluster solutions by screening out irrelevant features, when these numbers are not known in advance. However, this method suffers from the drawback that features need to belong to the same distribution family, which severely limits its application, because real data often include both numerical and categorical features. Further, its application is rather limited to low dimensional cases (*p* < *n*), because in high-dimensional cases, the number of objects to infer posterior distribution for the full covariance matrix of the Gaussian distribution may be insufficient, resulting in overfitting.

To address the aforementioned problems, we consider a multiple clustering framework in which we can make the best use of co-clustering structure that is not prone to overfitting. Concretely, we propose a novel multiple clustering method (referred to hereafter as the multiple co-clustering method) based on the following extension of the co-clustering model. First, we consider multiple views of co-clustering structure (Fig 1C), where a univariate distribution is fitted to each cluster block [21]. Second, for each cluster block, the proposed method simultaneously deals with an ensemble of several types of distribution families such as Gaussian, Poisson, and multinomial distribution (Fig 1D). Obviously, the first extension enables our model to fit high-dimensional data, while the second enables it to fit data that include different types of features (numerical and categorical). In particular, the second extension is quite novel, which allows one to simultaneously analyze a dataset of heterogeneous types of marginal distributions. To the best of our knowledge, such a multiple clustering method does not exist.

As an alternative approach, one may consider a multiple clustering model by simply fitting a univariate (mixture) distribution to each view (hereafter, we call it the ‘restricted multiple clustering method’). However, such an approach has the drawback that it may replicate similar object cluster solutions for different views. For instance, features that discriminate among object clusters in the same manner would be allocated to different views, if these are negatively correlated or if they have different scales (hence, redundant views). As a consequence, it would not only complicate interpretation, but would also lose discriminative power relative to features. In the present paper, we retain this method for performance comparisons with our method.

## Method

As in [19], our method is based on nonparametric mixture models using the Dirichlet process [22, 23]. However, unlike the conventional Dirichlet process, we employ a hierarchical structure, because in our model, the allocation of features is determined in two steps: the first allocation to a view, and the second to a feature cluster in that view. Moreover, we allow for mixing of several types of features, such as mixtures of Gaussian, Poisson, and categorical/multinomial distributions. Note that in this paper, we assume that types of features are pre-specified by the user, and do not draw inferences about them from data. In the following section, we formulate our method to capture these two aspects. To estimate model parameters, we rely on a variational Bayes EM (Expectation Maximization) algorithm, which provides (iterative) updating equations of relevant parameters. In general, determining whether these updating equations may be expressed in closed form is a subtle problem. However, this is the case in our model, which provides an efficient algorithm to estimate views and feature-/object cluster solutions. For notation used in this section, please refer to Table 2.

**Table 2:** Notation for multiple clustering model.

Domain	Notation	Description
Data	*n*	Sample size
	*m*	*m*^*th*^ distribution family (*m* = 1, …, *M*)
	*M*	Total number of distribution families
	*d*^(*m*)^	Number of features for distribution family *m*
	***X***^(*m*)^	Data matrix for distribution family *m* of size *n* × *d*^(*m*)^
	$X_{i}^{\left(m\right)}$	*i*^*th*^ sample for distribution family *m* of size 1 × *d*^(*m*)^
	***X***	All data matrix of size $n\times \sum_{m=1}^{M}d^{\left(m\right)}$
Cluster Membership	*V*	Number of views
	$G_{v}^{\left(m\right)}$	Number of feature clusters for distribution family *m* in view *v*
	*K*_*v*_	Number of object clusters in view *v*
	*G*^(*m*)^	$\underset{v}{\text{max}}\;G_{v}^{\left(m\right)}$
	*K*	$\underset{v}{\text{max}}\;K_{v}$
	***Y***^(*m*)^	Feature-partition indicators of size *d*^(*m*)^ × *V* × *G*^(*m*)^
	$Y_{j..}^{\left(m\right)}$	Feature-partition indicators for feature *j* of distribution family *m* of size *V* × *G*^(*m*)^
	$Y_{j,v,g}^{\left(m\right)}$	Element of ***Y***^(*m*)^: 1 if feature *j* of distribution family *m* belongs to cluster *g* in view *v*, or 0 otherwise
	***Z***	Object-partition indicators of size *n* × *V* × *K*
	***Z***_*i*, *v*._	Object-partition indicators for object *i* in view *v* of size 1 × *K*
	*Z*_*i*, *v*, *k*_	Element of ***Z***: 1 if object *i* belongs to object cluster *k* in view *v*, or 0 otherwise
Dirichlet Process	*w*_*v*_	Probability of stick-breaking for view *v*
	*α*_1_	Hypeparameter of a beta prior Beta(1, *α*_1_) for *w*_*v*_
	*π*_*v*_	Length of unit-stick ($\sum_{v=1}^{\infty }\pi_{v}=1$) for view *v*
	${w^{\prime }}_{g,v}^{\left(m\right)}$	Probability of stick-breaking for feature cluster *g* for distribution family *m* in view *v*
	*α*_2_	Hypeparameter of a beta prior Beta(1, *α*_2_) for ${w^{\prime }}_{g,v}^{\left(m\right)}$
	${\pi^{\prime }}_{g,v}^{\left(m\right)}$	Length of unit-stick ($\sum_{g=1}^{\infty }{\pi^{\prime }}_{g,v}^{\left(m\right)}=1$) for feature cluster *g* of distribution family *m* in view *v*
	$\tau_{g,v}^{\left(m\right)}$	$\pi_{v}{\pi^{\prime }}_{g,v}^{\left(m\right)}$: Length of unit-stick ($\sum_{g,v}^{\infty }\tau_{g,v}^{\left(m\right)}=1$) for feature cluster *g* of distribution family *m* in view *v*
	*u*_*k*, *v*_	Probability of stick-breaking for object cluster *k* in view *v*
	*β*	Hypeparameter of a beta prior Beta(1, *β*) for *u*_*k*, *v*_
	*η*_*k*, *v*_	Length of unit-stick ($\sum_{k=1}^{\infty }\eta_{k,v}=1$) for object cluster *k* in view *v*
Probability Model	$\theta_{v,g,k}^{\left(m\right)}$	Parameter(s) of distribution family *m* for feature cluster *g* and object cluster *k* in view *v*

### Multiple clustering model

We assume that a data matrix ***X*** consists of *M* distribution families that are known in advance. We decompose ***X*** = {***X***^(1)^, …, ***X***^(*m*)^, …, ***X***^(*M*)^} with data size *n* × *d*^(*m*)^ for ***X***^(*m*)^, where *m* is an indicator for a distribution family (*m* = 1, …, *M*). Further, we denote the number of views as *V* (common to all distribution families), the number of feature clusters $G_{v}^{\left(m\right)}$ for view *v* and distribution family *m*, and the number of object clusters *K*_*v*_ for view *v* (common to all distribution families). Moreover, for simplicity of notation, we use $G^{\left(m\right)}={\text{max}}_{v}G_{v}^{\left(m\right)}$ and *K* = max_*v*_ *K*_*v*_ to denote the number of features and the number of clusters, allowing for empty clusters.

With this notation, for i.i.d. *d*^(*m*)^-dimensional random vectors $X_{1}^{\left(m\right)},\ldots ,X_{n}^{\left(m\right)}$ for distribution family *m*, we consider a *d*^(*m*)^ × *V* × *G*^(*m*)^ feature-partition tensor (3rd-order) ***Y***^(*m*)^ in which $Y_{j,v,g}^{\left(m\right)}=1$ if feature *j* of distribution family *m* belongs to feature cluster *g* in view *v* (0 otherwise). Combining this for different distribution families, we let ***Y*** = {***Y***^(*m*)^}_*m*_. Similarly, we consider a *n* × *V* × *K* object-partition (3rd-order) tensor ***Z*** in which *Z*_*i*, *v*, *k*_ = 1 if object *i* belongs to object cluster *k* in view *v*. Note that feature *j* belongs to one of the views (i.e., $\sum_{v,g}Y_{j,v,g}^{\left(m\right)}=1$) while object *i* belongs to each view (i.e., $\sum_{k}Z_{i,v,k}^{\left(m\right)}=1$). Further, ***Z*** is common to all distribution families, which implies that our model estimates subject cluster solutions using information on all distribution families.

For a prior generative model of ***Y***, we consider a hierarchical structure of views and feature clusters: views are first generated, followed by generation of feature clusters. Thus, features are partitioned in terms of pairs of view and feature cluster memberships, which implies that the allocation of feature is jointly determined by its view and feature cluster. On the other hand, objects are partitioned into object clusters in each view, hence, we consider just a single structure of object clusters for ***Z***. We assume that these generative models are all based on a stick-breaking process as follows.

#### Generative model for feature clusters *Y*

We let $Y_{j\cdot \cdot }^{\left(m\right)}$ denote a view/feature cluster membership vector for feature *j* of distribution family *m*, which is generated by a hierarchical stick-breaking process:

$$\begin{array}{ccc} w_{v} & ∼ & \text{Beta}(\cdot |1,\alpha_{1}),\;v=1,2,\ldots \\ \pi_{v} & = & w_{v}\prod_{t=1}^{v-1}\left(1-w_{t}\right),\\ {w^{\prime }}_{g,v}^{\left(m\right)} & ∼ & \text{Beta}(\cdot |1,\alpha_{2}),\;g=1,2,\ldots ,m=1,\ldots ,M\\ {\pi^{\prime }}_{g,v}^{\left(m\right)} & = & {w^{\prime }}_{g,v}^{\left(m\right)}\prod_{t=1}^{g-1}\left(1-{w^{\prime }}_{t,v}^{\left(m\right)}\right),\\ \tau_{g,v}^{\left(m\right)} & = & \pi_{v}{\pi^{\prime }}_{g,v}^{\left(m\right)}\\ Y_{j\cdot \cdot }^{\left(m\right)} & ∼ & \text{Mul}(\cdot |\tau^{\left(m\right)}), \end{array}$$

where ***τ***^(*m*)^ denotes a 1 × *GV* vector $(\tau_{1,1}^{\left(m\right)},\ldots ,\tau_{G,V}^{\left(m\right)}{)}^{T}$ (the superscript *T* denotes matrix transposition); *Mul*(⋅|***π***) is a multinomial distribution of one sample size with probability parameter ***π***; *Beta*(⋅|*a*, *b*) is a Beta distribution with prior sample size (*a*, *b*); $Y_{j\cdot \cdot }^{\left(m\right)}$ is a 1 × *GV* vector $(Y_{j,1,1}^{\left(m\right)},\ldots ,Y_{j,V,G}^{\left(m\right)}{)}^{T}$. Note that we truncate the number of views with sufficient large *V* and the number of feature clusters with *G* [24]. When $Y_{j,v,g}^{\left(m\right)}=1$, feature *j* belongs to feature cluster *g* at view *v*. By default, we set the concentration parameters *α*_1_ and *α*_2_ to one.

#### Generative model for object clusters *Z*

A subject cluster membership vector of object *i* in view *v*, denoted as ***Z***_*i*, *v*⋅_, is generated by

$$\begin{array}{ccc} u_{k,v} & ∼ & \text{Beta}(\cdot |1,\beta ),\;v=1,2,\ldots ,\;k=1,2,\ldots \\ \eta_{k,v} & = & u_{k,v}\prod_{t=1}^{k-1}\left(1-u_{t,v}\right),\\ Z_{i,v\cdot } & ∼ & \text{Mul}(\cdot |\eta_{v}), \end{array}$$

where ***Z***_*i*, *v*⋅_ is a 1 × *K* (we take *K* sufficiently large) vector given by ***Z***_*i*, *v*⋅_ = (*Z*_*i*, *v*, 1_, …, *Z*_*i*, *v*, *K*_)^*T*^. We set the concentration parameter *β* to one.

**Fig 2:**
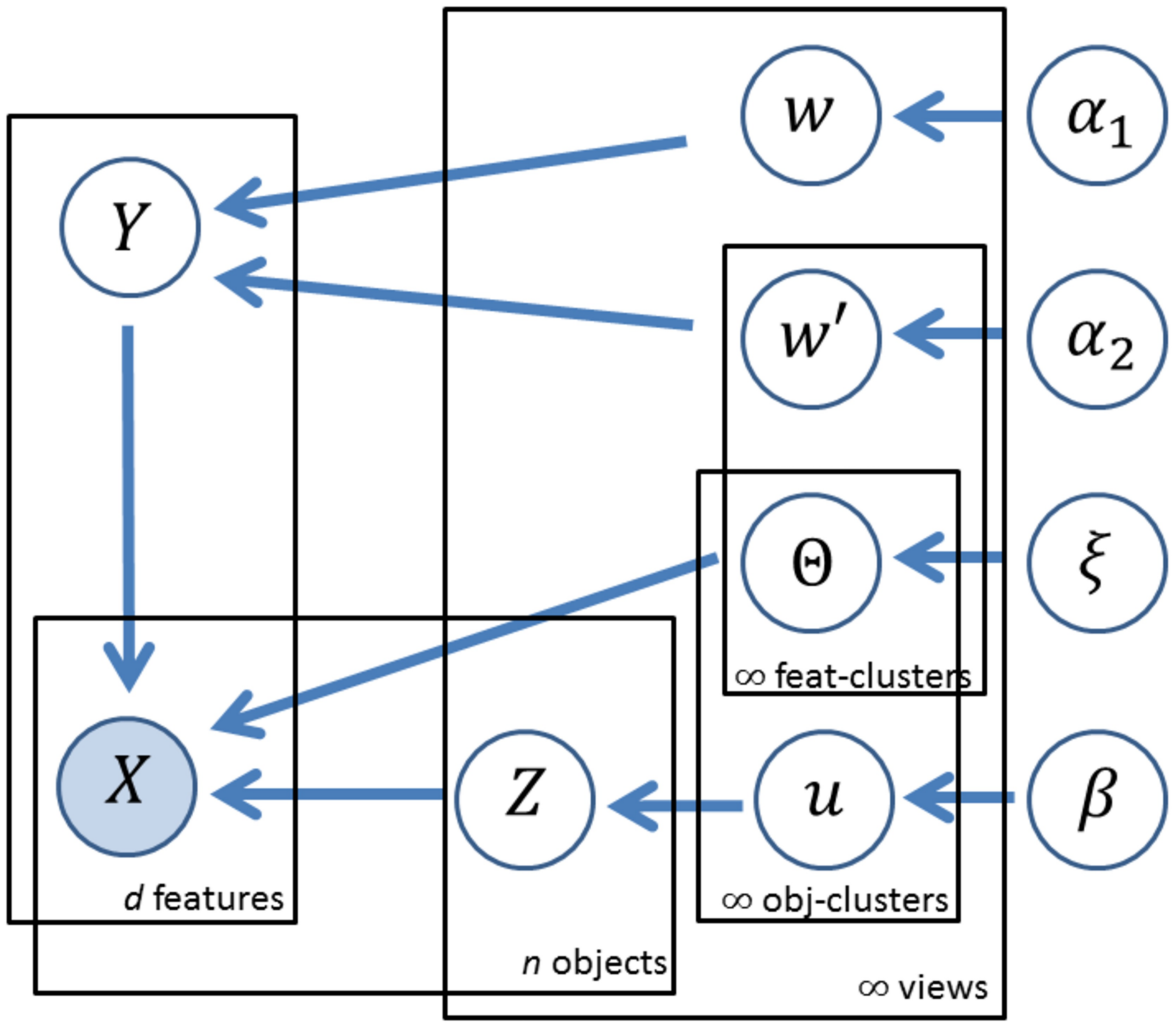
Graphical model of relevant parameters in our multiple co-clustering model. Feat- and obj-cluster denotes feature and object cluster, respectively. Note that *ξ* denotes all hyperparameters for distributions of parameters **Θ**.

Our multiple clustering model is summarized in a graphical model of Fig 2. It clarifies causal links among relevant parameters and a data matrix.

### Likelihood and prior distribution

We assume that each instance $X_{i,j}^{\left(m\right)}$ independently follows a certain distribution, conditional on ***Y*** and ***Z***. We denote $\theta_{v,g,k}^{\left(m\right)}$ as parameters of distribution family *m* in the cluster block of view *v*, feature cluster *g* and object cluster *k*. Further denoting $\Theta =\{\theta_{v,g,k}^{\left(m\right)}{\}}_{v,g,k,m}$, the logarithm of likelihood of ***X*** is given by

$$\begin{array}{ccc} & & \log p\left(X|Y,Z,\Theta \right)=\sum_{m,v,g,k,j,i}I\left(Y_{j,v,g}^{\left(m\right)}=1\right)I\left(Z_{i,v,k}=1\right)\log p\left(X_{i,j}^{\left(m\right)}|\theta_{v,g,k}^{\left(m\right)}\right), \end{array}$$

where I(x) is an indicator function, i.e, returning 1 if *x* is true, and 0 otherwise. Note that the likelihood is not directly associated with ***w*** = {*w*_*v*_}_*v*_, $w^{\prime }=\{{w^{\prime }}_{g,v}^{\left(m\right)}{\}}_{g,v}$ and ***u*** = {*u*_*k*, *v*_}_*k*, *v*_. The joint prior distribution of unknown variables ***ϕ*** = {***Y***, ***Z***, ***w***, ***w***′, ***u***, **Θ**} (i.e., class membership variables and model parameters) is given by

$$\begin{array}{c} p\left(w\right)p\left(w^{\prime }\right)p\left(Y|w,w^{\prime }\right)p\left(u\right)p\left(Z|u\right)p\left(\Theta \right). \end{array}$$

### Variational Inference

As in [19], we use variational Bayes EM for MAP (maximum a posteriori) estimation of ***Y*** and ***Z***. The logarithm of the marginal likelihood *p*(***X***) is approximated using Jensen’s inequality [25]:

$$\begin{array}{ccc} \log p(X) & \geq & \int q\left(\phi \right)\log\frac{p(X,\phi )}{q(\phi )}d\phi =L\left(q\left(\phi \right)\right), \end{array}$$ (1)

where *q*(***ϕ***) is an arbitrary distribution for parameters ***ϕ***. It can be shown that the difference between the left and right sides is given by the Kullback-Leibler divergence between *q*(***ϕ***) and *p*(***ϕ***|***X***), i.e., KL(q(ϕ),p(ϕ|X)). Hence, our approach of choosing *q*(***ϕ***) is to minimize KL(q(ϕ),p(ϕ|X)), which is tractable to evaluate. In our model, we choose *q*(***ϕ***) that is factorized over different parameters (mean field approximation):

$$\begin{array}{ccc} q(\phi ) & = & q_{w}\left(w\right)q_{w^{\prime }}\left(w^{\prime }\right)q_{Y}\left(Y\right)q_{u}\left(u\right)q_{Z}\left(Z\right)q_{\Theta }\left(\Theta \right), \end{array}$$

where each *q*(⋅) is further factorized over subsets of parameters, *w*_*v*_, ${w^{\prime }}_{g,v}^{\left(m\right)}$, $Y_{j\cdot \cdot }^{\left(m\right)}$, *u*_*k*, *v*_, ***Z***_*i*, *v*⋅_ and $\theta_{v,g,k}^{\left(m\right)}$.

In general, the distribution *q*_*i*_(*ϕ*_*i*_) that minimizes $\text{KL}(\prod_{l=1}^{L}q_{l}(\phi_{l}),p(\phi |X))$ is given by

$$\begin{array}{c} q_{i}\left(\phi_{i}\right)\propto \exp\left\{E_{-q_{i}\left(\phi \right)}\log p\left(X,\phi \right)\right\}, \end{array}$$

where $E_{-q_{i}(\phi )}$ denotes averaging with respect to ∏_*l* ≠ *i*_ *q*_*l*_(*ϕ*_*l*_) [26]. Applying this property to our model, it can be shown that

$$\begin{array}{ccc} q_{w}\left(w\right) & = & \prod_{v=1}^{V}\text{Beta}\left(w_{v}|\gamma_{v,1},\gamma_{v,2}\right)\\ q_{w^{\prime }}\left(w^{\prime }\right) & = & \prod_{m=1}^{M}\prod_{v=1}^{V}\prod_{g=1}^{G}\text{Beta}\left(w_{g,v}^{\left(m\right)}|\gamma_{g,v,1}^{\left(m\right)},\gamma_{g,v,2}^{\left(m\right)}\right)\\ q_{Y}\left(Y\right) & = & \prod_{m=1}^{M}\prod_{j=1}^{d^{\left(m\right)}}\text{Mul}\left(Y_{j\cdot \cdot }^{\left(m\right)}|\tau_{j}^{\left(m\right)}\right)\\ q_{u}\left(u\right) & = & \prod_{v=1}^{V}\prod_{k=1}^{K}\text{Beta}\left(u_{g,v}|\gamma_{k,v,1},\gamma_{k,v,2}\right)\\ q_{Z}\left(Z\right) & = & \prod_{v=1}^{V}\prod_{i=1}^{n}\text{Mul}\left(Z_{i,v\cdot }|\eta_{i,v}\right)\\ \log q_{\Theta }\left(\Theta \right) & = & \sum_{m,v,g,k,j,i}\tau_{j,v,g}^{\left(m\right)}\eta_{i,v,k}\log p\left(X_{i,j}^{\left(m\right)}|\theta_{v,g,k}^{\left(m\right)}\right)+\sum_{m,v,g,k}\log p\left(\theta_{v,g,k}^{\left(m\right)}\right)+\text{constant}, \end{array}$$

where the hyperparameters except for *q*_**Θ**_(**Θ**) are given by

$$\begin{array}{ccc} \gamma_{v,1} & = & 1+\sum_{m=1}^{M}\sum_{g=1}^{G}\sum_{j=1}^{d^{\left(m\right)}}\tau_{j,g,v}^{\left(m\right)}\\ \gamma_{v,2} & = & \alpha_{1}+\sum_{m=1}^{M}\sum_{t=v+1}^{V}\sum_{g=1}^{G}\sum_{j=1}^{d^{\left(m\right)}}\tau_{j,g,t}^{\left(m\right)}\\ \gamma_{g,v,1}^{\left(m\right)} & = & 1+\sum_{j=1}^{d^{\left(m\right)}}\tau_{j,g,v}^{\left(m\right)}\\ \gamma_{g,v,2}^{\left(m\right)} & = & \alpha_{2}+\sum_{t=g+1}^{G}\sum_{j=1}^{d^{\left(m\right)}}\tau_{j,t,v}^{\left(m\right)}\\ \gamma_{k,v,1} & = & 1+\sum_{i=1}^{n}\eta_{i,v,k}\\ \gamma_{k,v,2} & = & \beta +\sum_{t=k+1}^{K}\sum_{i=1}^{n}\eta_{i,v,t}\\ \log\tau_{j,g,v}^{\left(m\right)} & = & \sum_{k=1}^{K}\sum_{i=1}^{n}\eta_{i,v,k}E_{q(\theta )}[\log p(X_{i,j}^{\left(m\right)}|\theta_{v,g,k}^{\left(m\right)})]\\ & & +\psi \left(\gamma_{v,1}\right)-\psi \left(\gamma_{v,1}+\gamma_{v,2}\right)\\ & & +\sum_{t=1}^{v-1}\left\{\psi \left(\gamma_{t,2}\right)-\psi \left(\gamma_{t,1}+\gamma_{t,2}\right)\right\}\\ & & +\psi \left(\gamma_{g,v,1}^{\left(m\right)}\right)-\psi \left(\gamma_{g,v,1}^{\left(m\right)}+\gamma_{g,v,2}^{\left(m\right)}\right)\\ & & +\sum_{t=1}^{G-1}\left\{\psi \left(\gamma_{t,v,2}^{\left(m\right)}\right)-\psi \left(\gamma_{t,v,1}^{\left(m\right)}+\gamma_{t,v,2}^{\left(m\right)}\right)\right\}\\ & & +\text{constant}\\ \log\eta_{i,v,k} & = & \sum_{m=1}^{M}\sum_{g=1}^{G}\sum_{j=1}^{d^{\left(m\right)}}\tau_{j,g,v}^{\left(m\right)}E_{q(\theta )}[\log p(X_{i,j}^{\left(m\right)}|\theta_{v,g,k}^{\left(m\right)})]\\ & & +\psi \left(\gamma_{k,v,1}\right)-\psi \left(\gamma_{k,v,1}+\gamma_{k,v,2}\right)\\ & & +\sum_{t=1}^{K-1}\left\{\psi \left(\gamma_{t,v,2}\right)-\psi \left(\gamma_{t,v,1}+\gamma_{t,v,2}\right)\right\}\\ & & +\text{constant}, \end{array}$$ (2)

where $E_{q(\theta )}$ denotes averaging with respect to the corresponding *q*(***θ***) of $\theta_{v,g,k}^{\left(m\right)}$; *ψ*(⋅) denotes the digamma function defined as the first derivative of logarithm of gamma function. Note that $\tau_{j,g,v}^{\left(m\right)}$ is normalized over pairs (*g*, *v*) for each pair (*j*, *m*), while *η*_*i*, *v*, *k*_ normalized over *k* for each pair of (*i*, *v*). Observation models and priors of parameters **Θ** are specified in the following section.

### Observation models

For observation models, we consider Gaussian, Poisson, and categorical/multinomial distributions. For each cluster block, we fit a univariate distribution of these families with the assumption that features within the cluster block are independent. We assume conjugate priors for the parameters of these distribution families. Variational inference and updating equations are basically the same as in [19] (See S1 Appendix).

**Algorithm 1**. Variational Bayes EM for multiple co-clustering

**Input**: data matrices ***X***^(1)^, …, ***X***^(*M*)^.

**for** *s* = 1 **to** *S* **do**

 Randomly initialize {***τ***^(*m*)^}_*m*_ and {***η***_*v*_}_*v*_.

 **repeat**

  -Update the hyperparameters of relevant distribution families for *q*_**Θ**_(**Θ**).

  -Update the hyperparameters for *q*_***w***_(***w***), *q*_***w***′_(***w***′), *q*_***Y***_(***Y***), *q*_***u***_(***u***), and *q*_***Z***_(***Z***).

 **untill** *L* in Eq (3) converges.

 Keep *L*(*s*) = *L*

**end for**

s* = *argmax*_*s*_ *L*(*s*)

**Output**: MAP for ***Y*** and ***Z*** in the run s*.

### Algorithm

With the updating equations of the hyperparameters, the variational Bayes EM proceeds as follows. First, we randomly initialize {***τ***^(*m*)^}_*m*_ and {***η***_*v*_}_*v*_, and then alternatively update the hyperparameters until the lower bound L(q(ϕ)) in Eq (1) converges. This yields a locally optimal distribution *q*(***ϕ***) in terms of L(q(ϕ)). We repeat this procedure a number of times, and choose the best solution with the largest lower bound, as the approximated posterior distribution q*(***ϕ***). The MAP estimates of ***Y*** and ***Z*** are then evaluated as argmax_***Y***_ $q_{Y}^{*}(Y)$ and argmax_***Y***_ $q_{Z}^{*}(Z)$, respectively. The algorithm is outlined in Algorithm 1. Note that the lower bound L(q(ϕ)) is given by

$$\begin{array}{c} L(q(\phi ))=\int q(\phi )\log p(X|\phi )d\phi -\text{KL}(q(\phi ),p(\phi )), \end{array}$$ (3)

where both terms on the right side can be derived in closed form. It can be shown that this monotonically increases as *q*(***ϕ***) is optimized.

**Fig 3:**
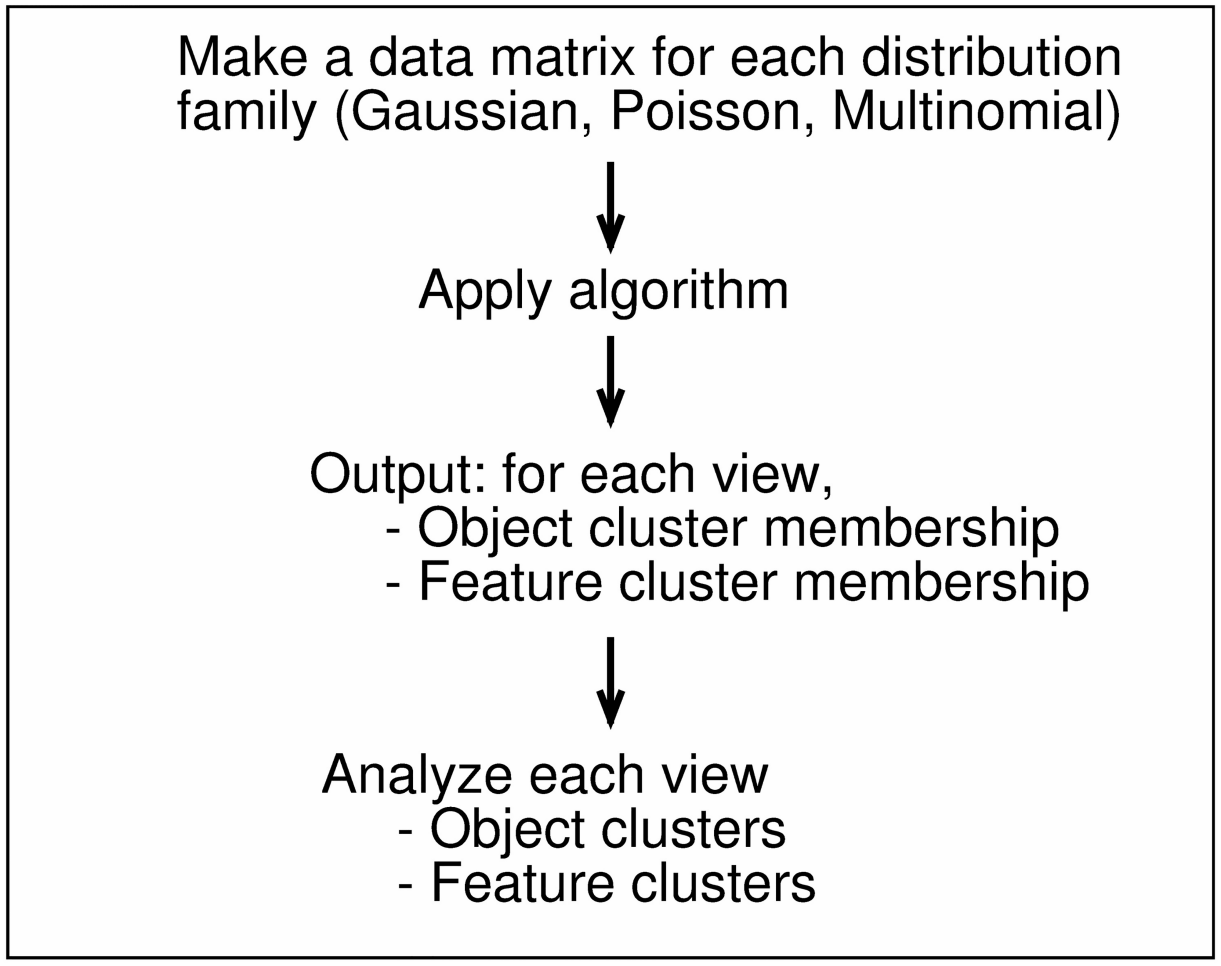
Flowchart for the proposed method. A user is required to identify a distribution family for a feature. For each distribution family, a data matrix is made. For Gaussian distribution, a feature is typically standardized. Application of the proposed method yields feature cluster memberships and object cluster membership in each view. This provides useful information on interpretation of view-wise cluster structures.

We illustrate a workflow of application of the proposed method in Fig 3. First, a user identifies a distribution family for each feature, generating a data matrix for the corresponding distribution family. Second, Algorithm 1 is applied to a set of these data matrices, which yields MAP estimates of ***Y*** and ***Z***. Third, using these estimates of ***Y*** and ***Z***, one analyzes object/feature cluster structures in each view.

### Time complexity

For simplicity, we consider time complexity of our algorithm for a single run. If we assume that the number of required iterations for convergence is the same, the time complexity of the algorithm is equivalent to the number of operations for updating the relevant parameters. In that case, as can be seen in the updating equations in Eq (2) and S1 Appendix, the time complexity is just *O*(*nd*) where *n* and *d* are the number of objects and the number of features (we fix the number of views and clusters). This enhances efficiency in applying our multiple co-clustering method to high-dimensional data. We return to this point later to compare other multiple clustering methods.

### Model representation

Our multiple co-clustering model is sufficiently flexible to represent different clustering models because the number of views and the number of feature-/object clusters are derived in a data-driven approach. For instance, when the number of views is one, the model coincides with a co-clustering model; when the number of feature clusters is one for all views, it matches the restricted multiple clustering model. Furthermore, when the number of views is one and the number of feature clusters is the same as the number of features, it matches conventional mixture models with independent features. Moreover, our model can detect non-informative features that do not discriminate between object clusters. In such a case, the model yields a view in which the number of object clusters is one. The advantage of our model is to automatically detect such underlying data structures.

### Missing values

Our multiple co-clustering model can easily handle missing values. Suppose that the missing entries occur at random, which may depend on the observed data, but not the missing ones (i.e., MAR, missing at random). We can deal with such missing values in a conventional Bayesian way, in which missing entries are considered as stochastic parameters [27]. In our model, this procedure is simply reduced to ignoring these missing entries when we update the hyperparameters. This is because (univariate) instances within a cluster block are assumed to be independent; hence the log-likelihood in Eq (1) is given by

$$\begin{array}{ccc} & & \log p(X^{obs}|Y,Z,\Theta )\\ & = & \sum_{m,v,g,k,j,i}I\left(Y_{j,v,g}^{\left(m\right)}=1\right)I\left(Z_{i,v,k}=1\right)I\left({\left(i,j\right)}^{\left(m\right)}\in o\right)\log p\left(X_{i,j}^{\left(m\right)}|\theta_{v,g,k}^{\left(m\right)}\right), \end{array}$$

where $I((i,j{)}^{\left(m\right)}\in o)$ is an indicator for the status of availability of the data cell of object *i* and feature *j* for distribution family *m* (1 when it is available, and 0 otherwise); ***X***^*obs*^ a subset of ***X*** that consists of the observed data only.

## Simulation study on synthetic data

In this section, we examine performance of our method in synthetic data. We consider both conventional and non-conventional settings of view structures.

### Conventional setting

**Fig 4:**
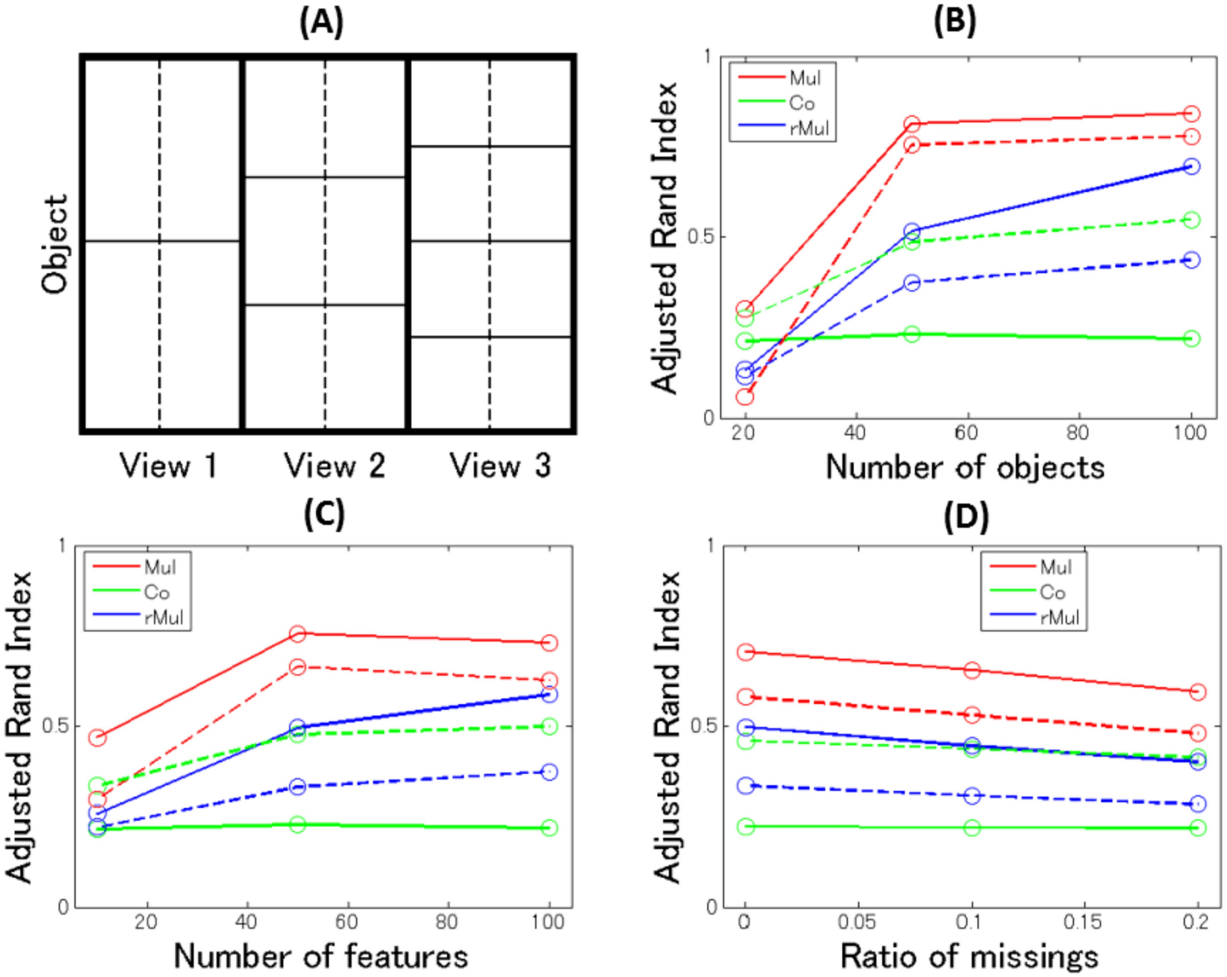
Data structure and results of simulation study on synthetic data in a conventional setting. Panel (A): Data structure for the simulation study. Each view has two feature clusters (separated by a dashed line) for each type of features of Gaussian, Poisson and Categorical. For Gaussian, means are set to (0, 4; 1, 3) ((0, 4) for top left and right cluster blocks, (1, 3) for bottom left and right cluster blocks) for view 1; (0, 5; 1, 4; 2, 3) for view 2; (0, 6; 1, 5; 2, 4; 3, 3) for view 3. The standard deviation is fixed to one. Similarly, for Poisson, the parameter *λ* is set to (1, 2; 2, 1), (1, 3; 2, 2; 3, 1), (1, 4; 2, 3; 3, 2; 4, 1). For categorical (binary), probability for success is (0.1, 0.9; 0.1, 0.9), (0.1, 0.9; 0.5, 0.5; 0.9, 0.1), (0.1, 0.9; 0.4, 0.6; 0.6, 0.4; 0.9, 0.1). Panels (B)-(D): Performance of the multiple co-clustering method (’Mul’, red), the co-clustering method (’Co’, green), and the restricted multiple clustering method (’rMul’, blue). Solid lines are for recovery of object cluster solutions, while dashed lines are for recovery of views. The results are summarized with respect to the number of objects (B), the number of features (C) and the proportions of missing entries (D).

In this subsection, we report on a simulation study to evaluate the performance of our method in a conventional setting. To the best of our knowledge, there is no algorithm in the literature that allows mixing of different types of features, as we have so far modeled. Hence, we compare the performance of our multiple co-clustering method only with co-clustering and restricted multiple clustering methods, which we model to accommodate different types of features. We set the hyperparameters *α*_1_, *α*_2_, and *β* relevant for generating views, feature clusters, and sample clusters to one, and the hyperparameters relevant for observations models to those specified in S1 Appendix. Note that we use this setting for further application of our method in the following sections. For data generation, we fixed the number of views to three and the number of object clusters to two, three, and four in views 1–3, respectively. The number of feature clusters was set to two in all views (Fig 4A). We manipulated the number of features (per view *and* distribution family) (10, 50, 100), the number of objects (20, 50, 100), and the proportion of (uniformly randomly generated) missing entries (0, 0.1, 0.2). We included three types of mixtures of distributions: Gaussian, Poisson, and Categorical. Memberships of views were evenly assigned to features for each distribution family, and the feature and object cluster memberships were uniformly randomly allocated. The distribution parameters for each cluster block were fixed as in the caption of Fig 4. We generated 100 datasets for each setting, which resulted in 100 × 27 = 2700 datasets.

We evaluated the performance of recovering the true cluster structure by means of an adjusted Rand index (ARI) [28]: When ARI is one, recovery of the true cluster structure is perfect. When ARI is close to zero, recovery is almost random. Specifically, we focused on recovery of memberships of views, and memberships of object clusters. Since the numbering of views is arbitrary, it is not straightforward to evaluate recovery of the true object cluster solutions (the correspondence between the yielded and the true object cluster solutions is not clear). Hence, to evaluate the performance of object cluster solutions, we first evaluated ARIs for all combinations of the true object clusters and yielded object cluster solutions, and then found the maximum ARI for each true object cluster solution. Lastly, we averaged the ARIs over views. In this manner, we evaluated the performance for the multiple co-clustering method and the restricted multiple clustering. The co-clustering method yields only a single object cluster solution; hence we averaged ARIs between the true object cluster solutions and this solution.

The performance of the multiple co-clustering method is reasonably good: performance of the recovery of views (red dashed line) and object clusters (red solid line) solutions improves as the number of objects increases (Table 3 and Fig 4B). Regarding the number of features, the performance improves as the number of features increases from 20 to 50, but there is no improvement from 50 to 100 (Fig 4C). This is possibly because in our simulation setting, each feature does not clearly discriminate between object clusters; hence, adding more features does not necessarily improve the recovery of views (hence, the recovery of object cluster solutions). Lastly, when the ratio of missing entries increases, the performance just becomes slightly worse, which suggests that our method is relatively robust to missing entries (Fig 4D).

**Table 3:** Summary of results of simulation study on synthetic data. Recovery of true object cluster structure and views evaluated in terms of mean values of adjusted Rand Index.

		Object clustering			Views		
Factors		Mul	Co	rMul	Mul	Co	rMul
Number of objects	20	**0.30**	0.21	0.13	0.05	**0.28**	0.11
	50	**0.81**	0.23	0.51	**0.75**	0.48	0.37
	100	**0.83**	0.22	0.69	**0.78**	0.54	0.43
Number of features	10	**0.47**	0.21	0.26	0.30	**0.33**	0.22
	50	**0.75**	0.22	0.49	**0.66**	0.47	0.32
	100	**0.74**	0.22	0.58	**0.63**	0.50	0.37
Ratio of missings	0	**0.71**	0.22	0.49	**0.58**	0.46	0.33
	0.1	**0.66**	0.22	0.44	0.53	0.44	0.30
	0.2	**0.60**	0.22	0.40	**0.48**	0.41	0.28

(a) Mul, Co, and rMul denote our multiple co-clustering, co-clustering and restricted multiple clustering methods.

(b) Digits denotes mean values of adjusted Rand Index over 9 × 100 = 900 datasets for a corresponding factor.

(c) To evaluate performance among three methods, we applied Friedman test, which is non-parametric equivalent of ANOVA. For significant cases at level of 0.01, we subsequently carried out Nemenyi test, which is non-parametric equivalent of the Tukey test. If the best performance among three methods is significant at level of 0.01 for this test, the corresponding digits are shown in bold.

As a whole, the multiple co-clustering method outperforms the co-clustering and the restricted multiple clustering methods (Table 3; we carried out Friedman test and Nemenyi test to statistically examine differences of performance among these methods [29]). The performance of the co-clustering method is poor because it does not fit the multiple clustering structure. On the other hand, the restricted multiple clustering method can potentially fit each object cluster structure; hence, it performs somewhat well in this regard (but, not for recovery of the true memberships of views).

### Non-conventional setting

In this subsection, we carry out simulation studies, which shed light on capability of our method in a non-conventional setting of data. First, we illustrate an example of application of our method to a dataset with a large number of views. Second, we illustrate an example of application for subspace clustering.

#### Large number of views

In this experiment, we consider synthetic data in which the true number of views is 20. The sample size of this data is 30 with 2000 features (each view consists of 100 features). For view *v* (*v* = 1, …, 19), two-object-cluster structure is assumed where one cluster consists of 15 samples. We generated the first 15 samples from a normal distribution with mean (2*v* − 1) and standard deviation 0.1, while the remainder of 15 samples from a normal distribution with mean 2*v* and standard deviation 0.1. In this way, we independently generated samples for 100 features in each view. To differentiate object-cluster membership for each view, we randomized the order of samples for each view. For the 20th view, we generated all samples from the standard normal distribution. To make obvious differences among views, we did not standardize a dataset. We randomly generated 100 datasets of this kind, which were subsequently applied by our method.

To evaluate the performance, we focus on the number of perfectly recovered views (Table 4). It is shown that view memberships in six to twelve views were correctly identified without errors. However, taking into account that the true views are clearly separated, the performance is not necessarily impressive. A possible reason for the insufficient performance of our method is that search space for cluster solutions is considerably large, which reveals limitations of our method.

**Table 4:** Results on recovery of views for data with a large number of views.

Number of perfectly recovered views (out of 20)	Proportion of datasets
6	4%
7	6%
8	14%
9	31%
10	13%
11	26%
12	6%

Nevertheless, further application of our method to a subset of data may correctly recover all views. In all datasets, it was found that a couple of the true views were simply merged into a single view yielded by our method. This suggests a possibility that we may recover the true views by re-applying our method to a subset of data consisting of the selected features in each view. A desired scenario is that in the re-application, the merged view splits into the true views while non-merged view does not.

#### Subspace clustering

**Fig 5:**
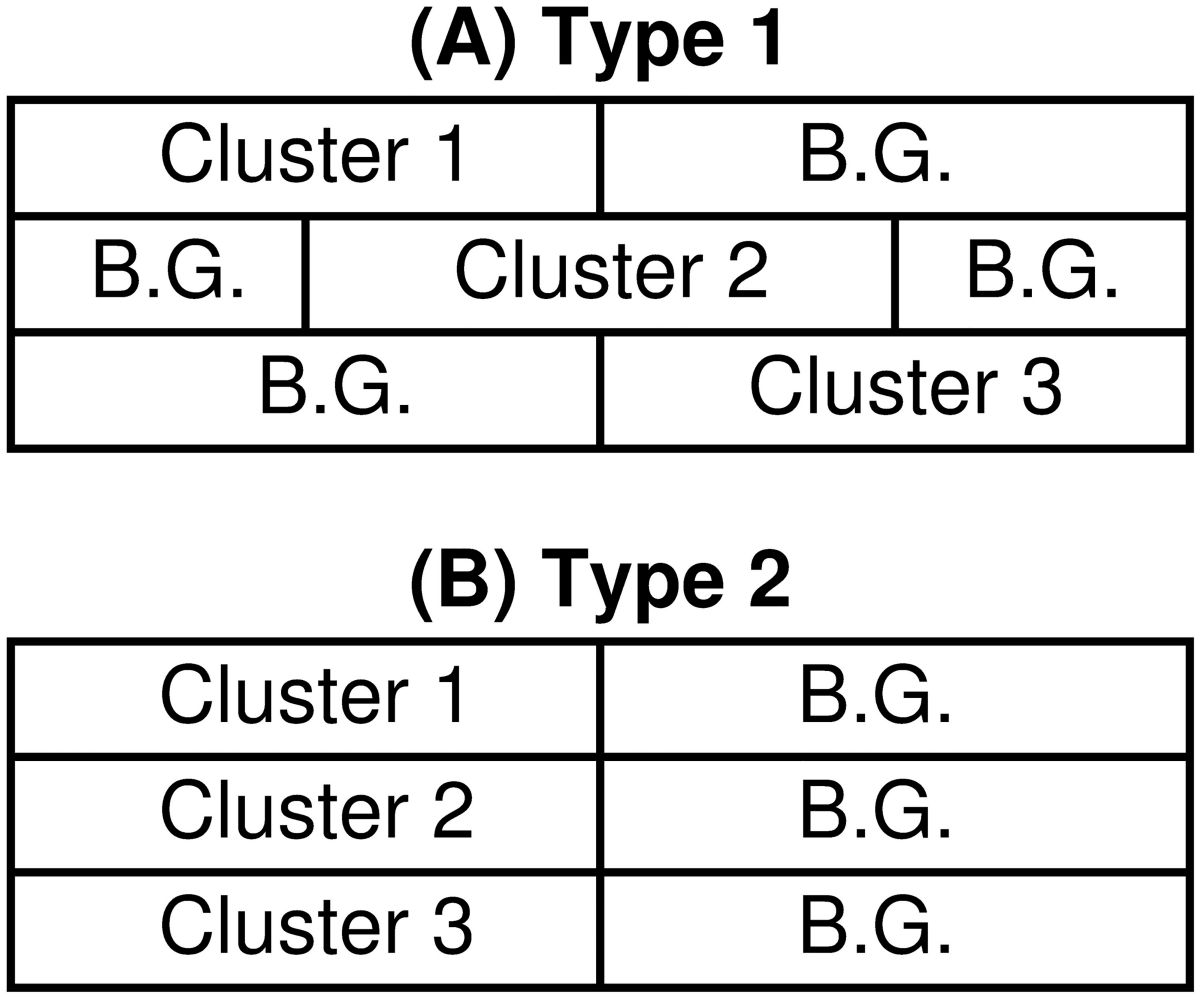
Configurations of data matrix. In these illustrations, the horizontal axis denotes features while the vertical axis objects. Cluster blocks are denoted by Cluster 1, 2, and 3, while background entries are denoted by B.G. For panel (A), clusters are embedded in different subspace (Type 1), while for panel (B), clusters are in the same subspace (Type 2).

Next, we consider an experiment for subspace clustering. In this setting, clusters are embedded in different feature-subspace (Fig 5A) [30–32]. On the other hand, our proposed algorithm assumes that object clusters are in the same subspace of a given view (Fig 5B). Hence, our method does not suit the assumption of subspace clustering. Nonetheless, it is an interesting question how our method works in such a non-conventional setting.

We compare performance of our method with a benchmark method in this domain: An entropy weighting *K*-means algorithm (Erwk) [33]. This algorithm was specifically designed for subspace clustering, hence, it is expected that Erwk may outperform our method. On the other hand, for datasets in which clusters are embedded in the same subspace, our method may outperform Erwk.

In this simulation study, we consider two types of datasets. One is a typical subspace structure (Type 1) in which clusters are embedded in different subspace (Fig 5A). On the other hand, we also consider a special subspace structure (Type 2) in which clusters are embedded in the same subspace (Fig 5B). Type 2 data can be considered as a two-view structure: a first view discriminates between clusters; a second view does not (i.e., consisting of background features). In both cases, we set data size to 300 × 12 (sample size 300 and the number of features 12); the number of clusters to three with cluster size 100; the number of relevant features to four for each cluster. Samples are generated from normal distributions where we fixed means for three clusters to 0, 2, and 3 while manipulating precision (reciprocal of variance) to 1, 4, 100 and 10000. For background features, each entry is generated by the standard normal distribution. For each setting, we generated 100 datasets. So, the total number of datasets is 2(*types*) × 4(*precision*) × 100 = 800.

**Fig 6:**
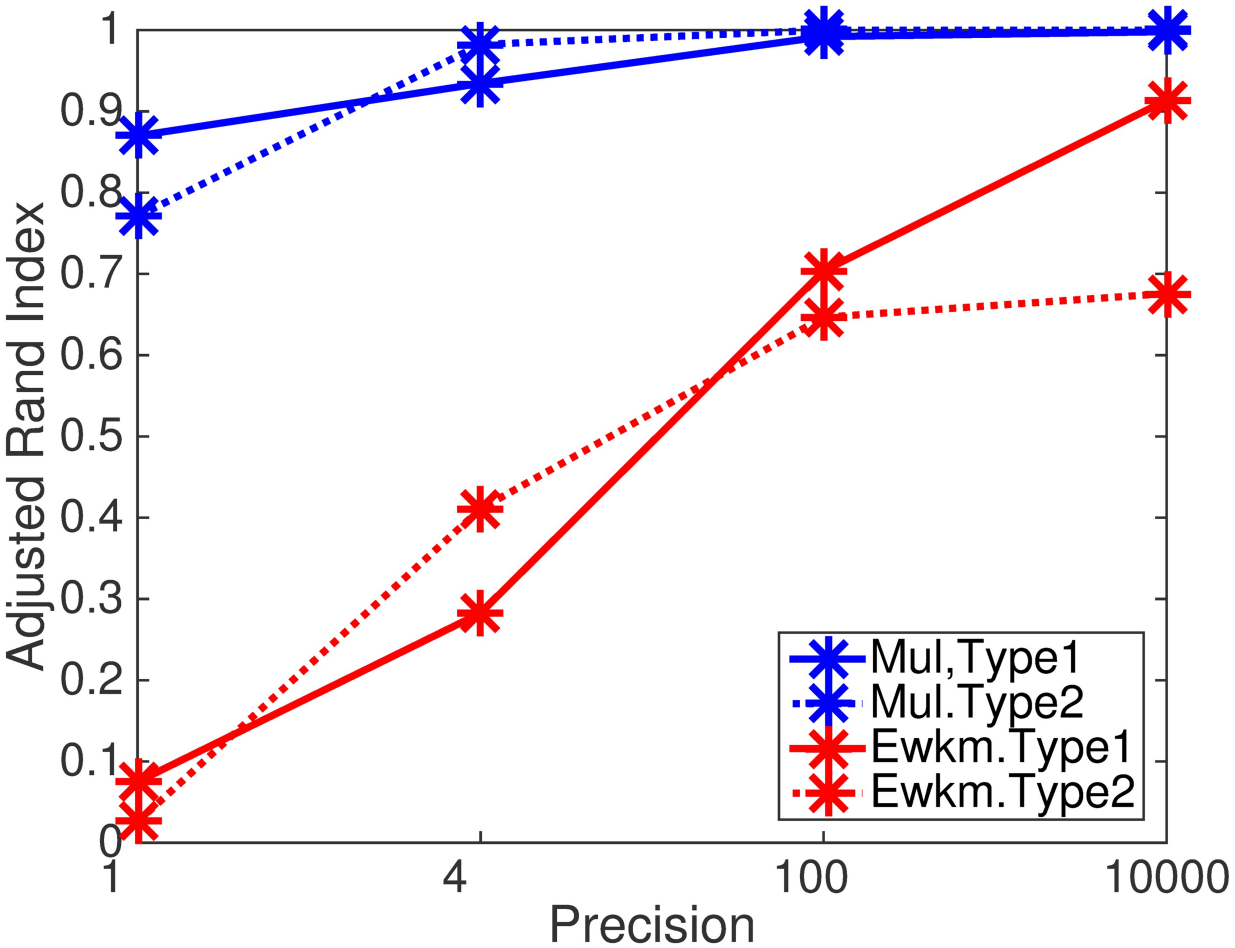
Results of simulation study of our method and Ewkm (we used R package ‘wskm’) on subspace clustering. Recovering of the true cluster structure measured by average Adjusted Rand Index.

We evaluated the performance of the recovering of the true object cluster structure by means of Adjusted Rand Index. The simulation study suggests that our method outperforms Erwk (Fig 6, Table 5) both in Type 1 and Type 2 configurations.

**Table 5:** Results of simulation study of our method and Ewkm (we used R package ‘wskm’) on subspace clustering. Recovering of the true cluster structure measured by average Adjusted Rand Index. Bold digits denote significance at 0.01 level by Wilcoxon signed-rank test on differences of performance between two methods.

Type of datasets	Precision	Methods	
		Multiple co-clustering	Ewkm
Type 1 (Subspace str.)	1	**0.86**	0.07
	4	**0.93**	0.28
	100	**0.99**	0.70
	10000	**0.99**	0.91
Type 2 (Non-subspace str.)	1	**0.77**	0.02
	4	**0.98**	0.41
	100	**0.99**	0.64
	10000	**0.99**	0.67

A close analysis on the clustering results reveals a possible reason for the unexpected good performance of our method in Type 1 datasets. The key of success lies in feature clusters. Though any feature does not perfectly discriminate the given three clusters, a combination of them does so. For instance, feature 1–3 distinguishes cluster 1 from the remainder, while feature 10–12 does cluster 3. So, a view that contains both features 1–3 and 10–12 can potentially discriminate three clusters. Though it may be too optimistic to expect our method to always work in this way, this experiment shows some workability of our method in subspace clustering.

## Application to real data

To test our multiple co-clustering method on real data, we consider three datasets: facial image data, cardiac arrhythmia data, and depression data. For facial image and cardiac arrhythmia data, the (possible) true sample clustering label is available, which enables us to evaluate clustering performance of our multiple co-clustering method. We compare the performance with the restricted multiple clustering method and two state-of-the-art multiple clustering methods: the constrained orthogonal average link algorithm (COALA, [13]) and the decorrelated *K*-means algorithm [16]. These state-of-the-art methods aim to detect dissimilar multiple sample clustering solutions without partitioning of features. COALA is based on a hierarchical clustering algorithm, while decorrelated *K*-means is based on a *K*-means algorithm. The two methods also differ in the way to detect views: COALA iteratively identifies views while decorrelated *K*-means simultaneously does so. For both methods, we need to set the number of views and the number of sample clusters. In this experiment, we set these to the (possible) true numbers. For the depression data, no information is available on true cluster structure. Hence, we focus mainly on implications drawn from the data by our multiple co-clustering method, rather than evaluating the performance of recovery of true cluster structure.

### Facial image data

**Fig 7:**
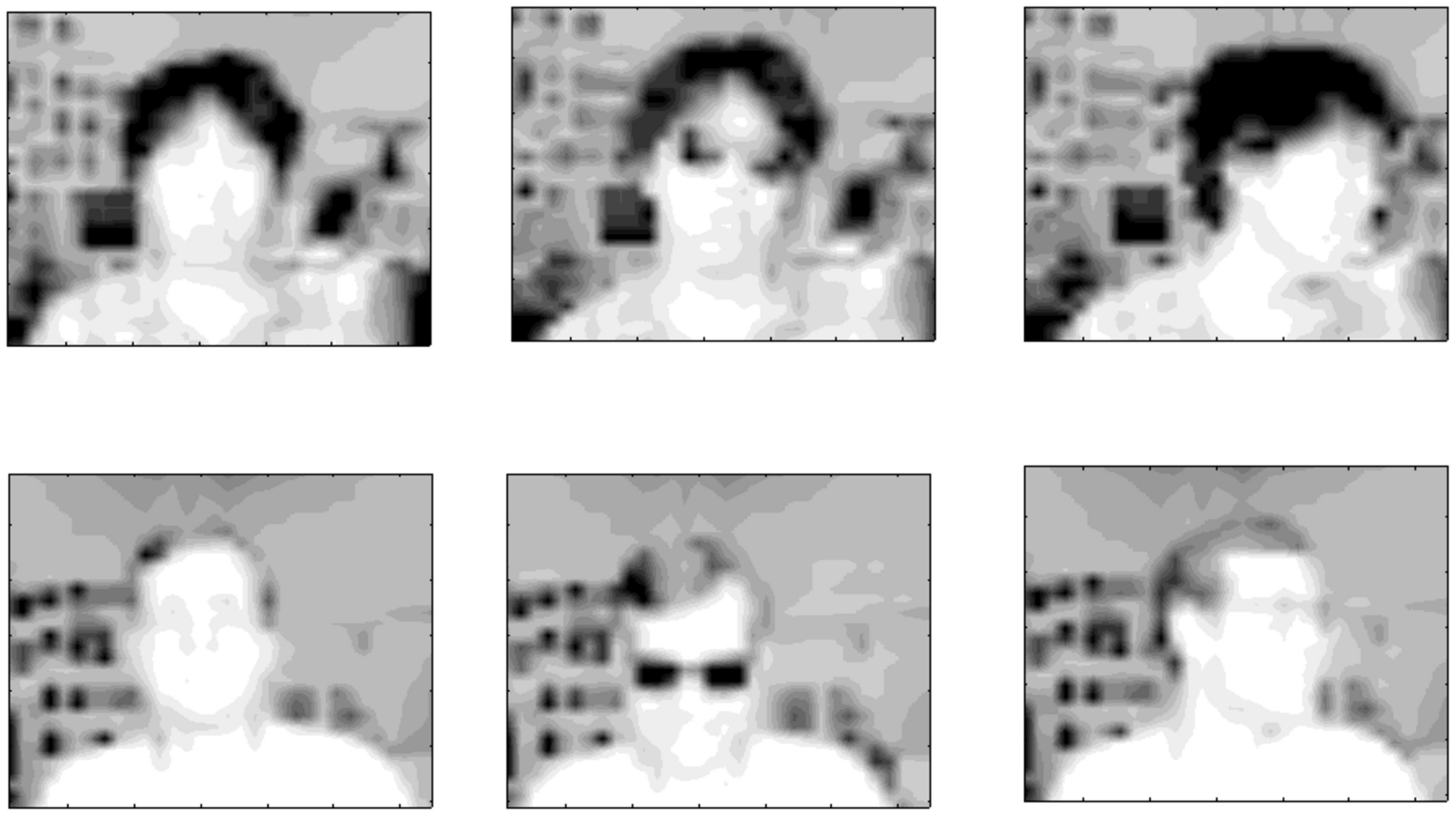
Samples from the facial image data. The first row represents person ‘an2i’ with configurations of (no sunglasses, straight pose and neutral expression), (sunglasses, straight pose, angry expression) and (no sunglasses, left pose, happy expression) from left to right columns. The second row for person ‘at33’ with the same patterns of configuration.

The first dataset contains facial image data from the UCI KDD repository (http://archive.ics.uci.edu/ml/datasets.html), which consists of black and white images of 20 different persons with varying configurations (Fig 7): *eyes* (wearing sunglasses or not), *pose* (straight, left, right, up), and *expression* (neutral, happy, sad, angry). This dataset served as a benchmark for multiple clustering in several papers [13, 19]. Here, we focus on the quarter-resolution images (32 × 30) of this dataset, which results in 960 features. For simplicity, we consider two subsets of these images: data 1 consisting of a single person (named ‘an2i’) with varying *eyes*, *pose* and *expression* (data size: 32 × 960); data 2 consisting of two persons (in addition to ‘an2i’, we include person ‘at33’, data size: 64 × 960). We use these datasets without pre-processing.

The facial image data has multiple clustering structures that can be characterized by all of the features (global) or some of the features (local). Identification of persons (hereafter, *useid*) may be related to global information of the image (all features), while *eyes*, *pose* and *expression* are based on local information (a subset of features). Here, we focus only on *pose*, which is a relatively easy aspect to detect [34]. Since COALA and decorrelated *K*-means methods do not explicitly model relevant features for sample clustering, they can potentially capture a multiple clustering structure based on either global or local information of such a dataset. On the other hand, our multiple co-clustering model is based on a partition of features, which implicitly assumes that a possible sample clustering structure is based on non-overlapping local information. Our interest in this application is to examine the performance of our multiple clustering method using such data.

To evaluate performance, we focus only on sample clustering solutions. We base our evaluation criterion on recovery of structures of *useid* and *pose* (*useid* is applicable only for Data 2), which is measured by the maximum value of an adjusted Rand Index between the true sample structure in question and resulting sample clustering solutions. We discuss the results for each data in the following sub-sections.

#### Results: Data 1

**Fig 8:**
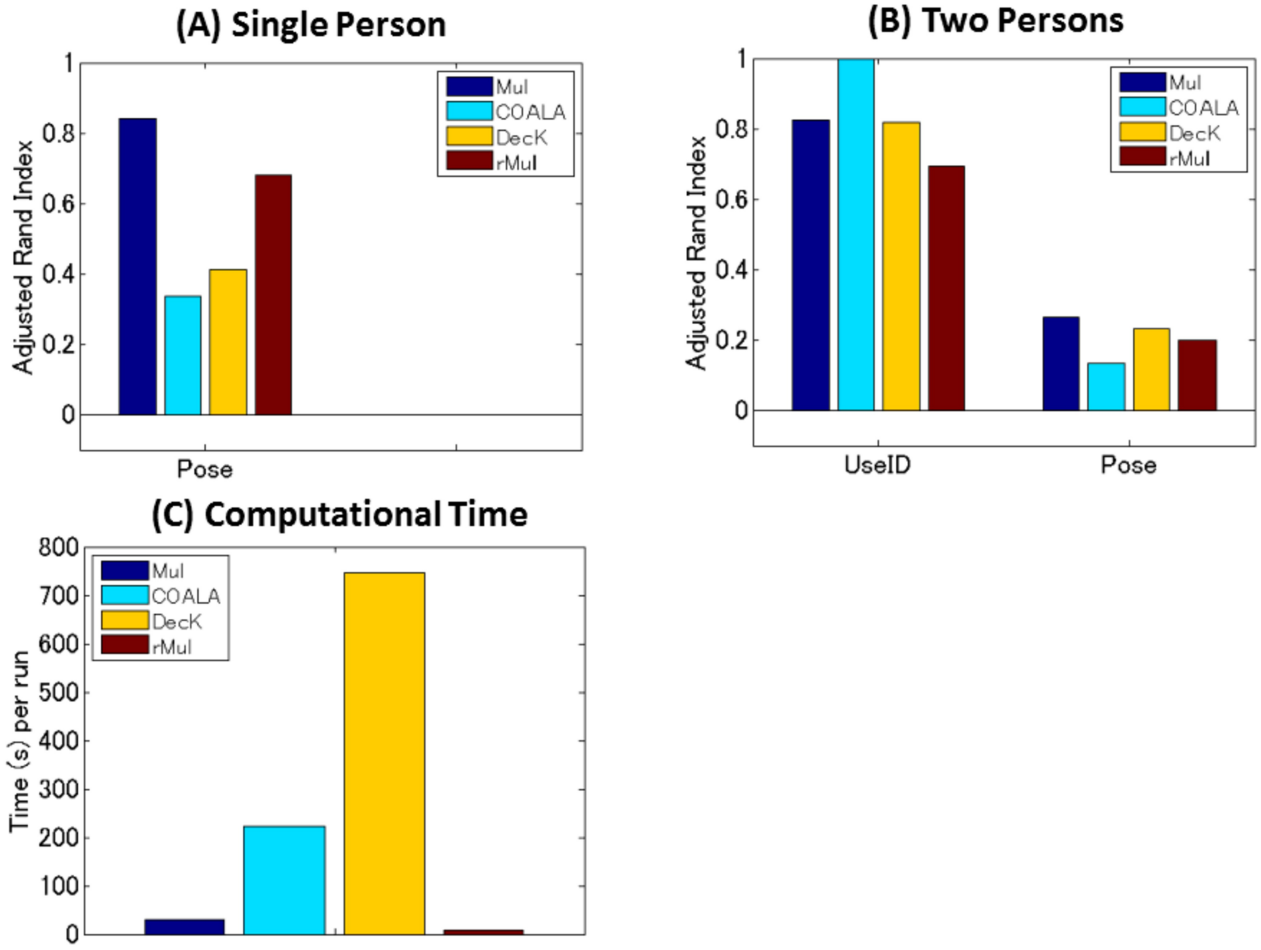
Performance on sample clusterings for the facial image data. Panel (A) for the subset (Data 1) of a single person (‘an2i’). Performance on pose for four clustering methods, i.e., multiple co-clustering (Mul), COALA, decorrelated *K*-means (DecK), and restricted multiple clustering (rMul) are evaluated by adjusted Rand Index of sample clustering solutions. Panel (B) for the subset (Data 2) of two persons (‘an2i’ and ‘at33’). Performance is evaluated on useid and pose. Note that to match true and yielded views, we evaluated the maximum value of adjusted Rand index between the true sample clustering in question and the yielded sample cluster solutions. The number of initializations is 500 for the multiple co-clustering, decorrelated *K*-means and the restricted multiple clustering. Panel (C) for computation time (seconds) per single run of each clustering method.

Our multiple co-clustering method yielded nine sample clusterings (i.e., nine views), one of which is closely related to *pose* with an adjusted Rand Index of 0.84 (Fig 8A, *p* <0.001 by permutation test, and Table 6 for the contingency table between true clusters and resultant sample clusters). Our method outperforms COALA and decorrelated *K*-means methods (the performance of both methods is similar), and performs slightly better than the restricted multiple clustering method.

**Table 6:** Results of sample clustering for data 1 of the facial image data. Contingency table of the true labels (pose) and yielded clusters of multiple co-clustering (Mul), COALA, decorrelated *K*-means (DecK), and restricted multiple (rMul) method from (a) to (d). T1, T2, T3 and T4 are true classes of pose (straight, left, right and up); C1, C2, C3, C4 and C5 are yielded clusters for each method.

(a) Mul					(b) COALA				
	**T1**	**T2**	**T3**	**T4**		**T1**	**T2**	**T3**	**T4**
**C1**	8	0	0	2	**C1**	2	0	6	1
**C2**	0	8	0	0	**C2**	6	0	1	7
**C3**	0	0	8	0	**C3**	0	5	1	0
**C4**	0	0	0	6	**C4**	0	3	0	0
(c) DecK					(d) rMul				
	**T1**	**T2**	**T3**	**T4**		**T1**	**T2**	**T3**	**T4**
**C1**	0	0	2	2	**C1**	0	8	0	0
**C2**	2	3	1	2	**C2**	0	0	8	0
**C3**	4	4	4	3	**C3**	1	0	0	5
**C4**	2	1	1	1	**C4**	4	0	0	2
					**C5**	3	0	0	1

**Fig 9:**
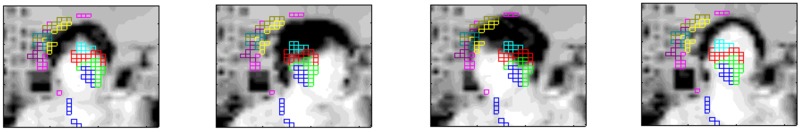
Selected features by our multiple co-clustering method for person ‘an2i’ in the facial image data. Pixels surrounded by color boxes are the selected features that yielded the relevant sample clustering to pose. Color denotes a particular feature cluster.

Next, we analyze features that are relevant to the sample clustering based upon *pose*. Note that our multiple co-clustering method yields information about features relevant to a particular sample clustering solution in an explicit manner while COALA and decorrelated *K*-means do not. Our method yielded the pixels (features) relevant to the cluster assignment, concentrating around subregions in the right part of head and the left part of face (Fig 9). This allows us to conclude that these subregions are very sensitive to different poses.

#### Results: Data 2

Our multiple co-clustering method yielded ten sample clusterings, three of which were closely related to *useid* (identification of person) and *pose* with adjusted Rand Indices of 0.82 (*p* <0.001) and 0.26 (*p* <0.001), respectively (Fig 8B, and Tables 7 and 8 for the contingency tables for *useid* and *pose*, respectively). To compare with COALA, our multiple co-clustering method performed a bit poorly for detecting *useid*, while it performed better for *pose*. On the other hand, the performance of our method is comparable to the decorrelated *K*-means method. Further, it performed slightly better than the restricted multiple clustering method.

**Table 7:** Results for data 2 of the facial image data. Contingency table of the true labels (useid) and yielded clustering of the multiple co-clustering (Mul), COALA, decorrelated *K*-means (DecK), and restricted multiple (rMul) method from (a) to (d). T1 and T2 are true classifications (an2i, at33); C1, C2, C3 and C4 are yielded clusters.

(a) Mul			(b) COALA		
	**T1**	**T2**		**T1**	**T2**
**C1**	32	0	**C1**	32	0
**C2**	0	25	**C2**	0	32
**C3**	0	7			
(c) DecK			(d) rMul		
	**T1**	**T2**		**T1**	**T2**
**C1**	3	32	**C1**	32	0
**C2**	29	0	**C2**	0	15
			**C3**	0	13
			**C4**	0	4

**Table 8:** Results of sampling-clustering for data 2 of the facial image data. Contingency table of the true labels (pose) and yielded clustering of the multiple co-clustering (Mul), COALA, decorrelated *K*-means (DecK), and restricted multiple (rMul) method from (a) to (d). T1, T2, T3 and T4 are true classes of pose (straight, left, right and up); C1, …, C7 are yielded results for each method.

(a) Mul					(b) COALA				
	**T1**	**T2**	**T3**	**T4**		**T1**	**T2**	**T3**	**T4**
**C1**	7	8	0	8	**C1**	8	1	1	6
**C2**	1	0	0	8	**C2**	0	7	0	0
**C3**	0	1	7	0	**C3**	0	0	9	0
**C4**	0	7	1	0	**C4**	8	8	6	10
**C5**	7	0	0	0					
**C6**	0	0	5	0					
**C7**	1	0	3	0					
(c) DecK					(d) rMul				
	**T1**	**T2**	**T3**	**T4**		**T1**	**T2**	**T3**	**T4**
**C1**	7	4	0	2	**C1**	5	3	2	4
**C2**	0	3	14	3	**C2**	5	0	8	0
**C3**	6	3	0	10	**C3**	0	0	6	4
**C4**	3	6	2	1	**C4**	1	1	0	8
					**C5**	2	7	0	0
					**C6**	3	5	0	0

**Fig 10:**
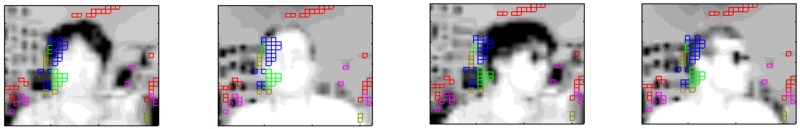
Samples from image datasets for person ‘an2i’ and ‘at33’. Pixels surrounded by color boxes are selected features that yielded relevant sample clustering to useid in data2. Image configurations are (‘an2i’, non sunglass, straight), (‘at33’, non sunglass, straight), ‘an2i’, sunglass, left), and (‘at33’, sunglass, left), respectively. Expression is neutral for all samples. In these examples, the multiple clustering method correctly identified these persons.

The most relevant pixels for *useid* concentrate near the right part of face, and the background (Fig 10). This can be interpreted to mean that the difference in hair style may be an important factor to distinguish between these two persons. In addition, an apparent difference in their rooms (background) also serves as a discriminating factor.

### Cardiac arrhythmia data

Next, we apply our multiple co-clustering method to Cardiac Arrhythmia data (UCI KDD repository). Unlike the facial image data in the previous section, this dataset does not necessarily have multiple sample clustering structures (indeed, such information is not available). However, the multiple co-clustering method should be able to automatically select relevant features.

The original dataset consisted of 452 samples (subjects) and 279 features that comprise various cardiac measurements and personal information such as sex, age, height, and weight (See more detail in [35]). Some of these features are numerical (206 features) and others are categorical (73 features). Further, there are a number of missing entries in this dataset. Beside these features, a classification label is available, which classifies the subjects into one of 16 types of arrhythmia. For simplicity, we focus only on three types of arrhythmia of similar sample size: *Old Anterior Myocardial infarction* (sample size 15), *Old Inferior Myocardial Infarction* (15) and *Sinus Tachycardia* (13). The objective in this section is to examine recovery performance among these three types of arrhythmia and identify relevant features for these subtypes.

Application of COALA and decorrelated *K*-means methods to this dataset is problematic because these methods do not allow for categorical features nor missing entries. Hence, we use the following heuristic procedure to pre-process the data: Re-code a binary categorical feature using a numerical feature (taking values 0 or 1); replace missing entries with mean values of features. Recall that these problems do not arise with our multiple co-clustering method.

#### Results

**Fig 11:**
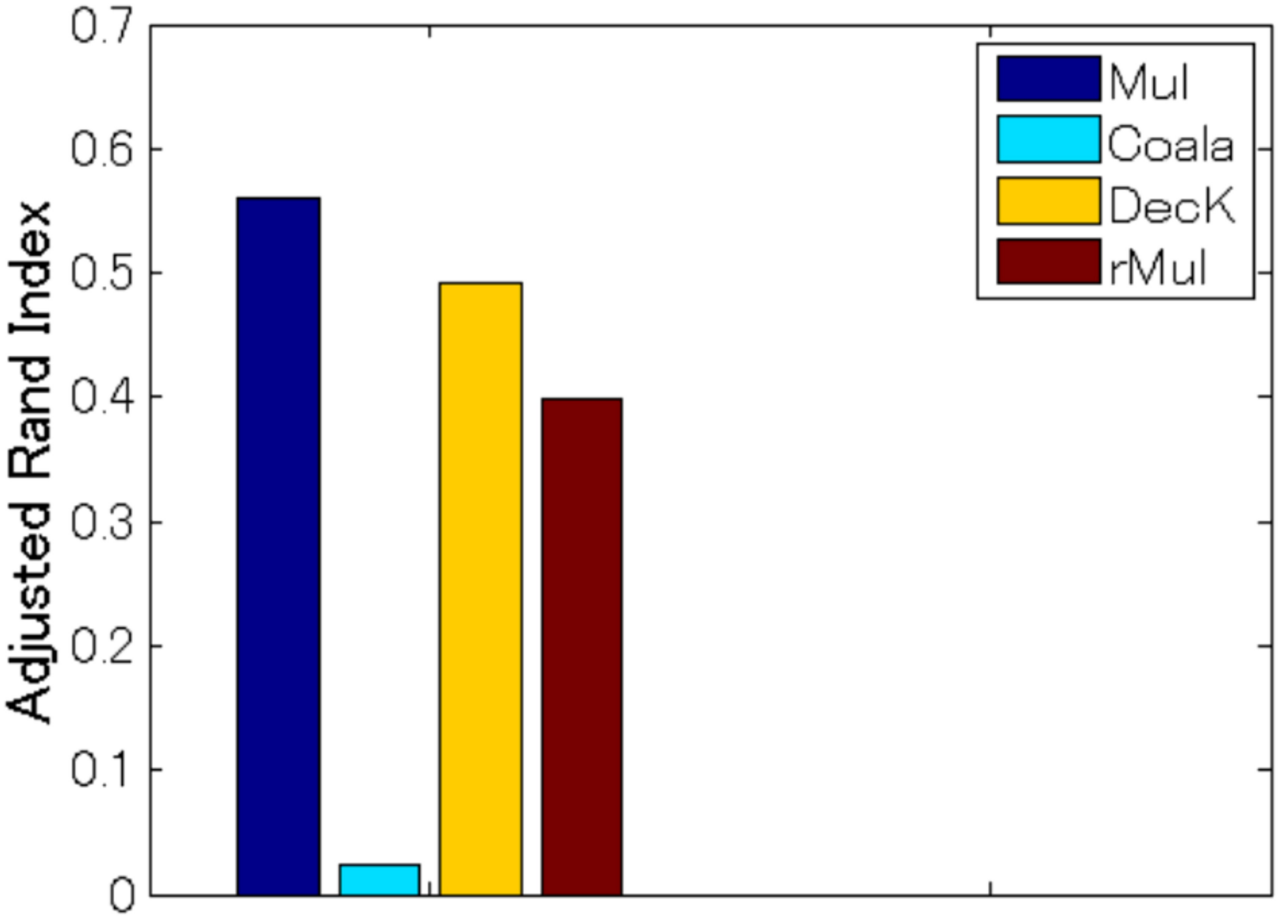
Results of multiple clustering for the cardiac arrhythmia data. Comparison of performance on subject clustering in terms of adjusted Rand Index among multiple co-clustering, COALA, decorrelated *K*-means and restricted multiple clustering methods.

Our multiple co-clustering method yielded nine sample clusterings (i.e., nine views). The maximum adjusted Rand Index between the true labels and resultant clusters is 0.56 (*p* <0.001, Fig 11). On the other hand, the maximum Rand Index for COALA, decorated *K*-means and restricted multiple clustering methods are 0.02, 0.49 and 0.39, respectively.

Further, we examine subject clustering results more in detail. For our multiple co-clustering method, the subject clusters C1, C2, and C3 distinguish the three symptoms well (corresponding to T2, T1, and T3, respectively, Table 9). On the other hand, such a distinction is totally or partially ambiguous for the other methods. In the case of COALA, clustering results seem to be degenerate, yielding two tiny clusters (C2 and C3). For decorrelated *K*-means, the distinction among T1, T2, and T3 is partially ambiguous. There is a clear correspondence between C1 and T1, but C2 is a tiny cluster, and C3 does not distinguish between T2 and T3. A similar observation is made for the restricted multiple clustering method.

**Table 9:** Results of sample clustering for the cardiac arrhythmia data. Contingency table of the true labels and yielded clustering of the multiple co-clustering (Mul), decorrelated *K*-means (DecK), and restricted multiple (rMul) method from (a) to (d). T1, T2, and T3 are true classes of arrhythmia (Old Anterior Myocardial Infarction, Old Inferior Myocardial infarction, and Sinus Bradycardy, respectively); C1, C2, C3 and C4 are yielded results for each method.

(a) Mul				(b) COALA			
	**T1**	**T2**	**T3**		**T1**	**T2**	**T3**
**C1**	0	14	6	**C1**	15	15	10
**C2**	14	0	0	**C2**	0	0	2
**C3**	1	1	5	**C3**	0	0	1
**C4**	0	0	2				
(c) DecK				(d) rMul			
	**T1**	**T2**	**T3**		**T1**	**T2**	**T3**
**C1**	14	0	0	**C1**	14	0	2
**C2**	0	0	1	**C2**	1	5	4
**C3**	1	15	12	**C3**	0	3	6
				**C4**	0	7	1

Finally, we examine selected features for the relevant clustering solution by our multiple co-clustering method. For the numerical features, 98 out of 205 features were selected while all categorical features were selected. Detailed analysis on these selected features may require medical expertise on electrocardiogram, which is beyond the scope of this paper. On the other hand, for non electrocardiogram features such as sex, age, height, weight and heart rate (the number of heart beats per minute), only sex was selected. In particular, the result that heart rate was not selected for these subtypes of heart disease suggests that these symptoms were not distinguishable simply by heart rate. Hence, clinical examination of electrical activity of the heart becomes essential. Note that other methods such as COALA and decorrelated *K*-means methods do not select features, hence, feature analysis is not possible for these methods.

### Depression data

Lastly, we apply our multiple co-clustering method to depression data, which consists of clinical questionnaires and bio-markers for healthy and depressive subjects. This study was approved by the Research Ethics Committee at the Okinawa Institute of Science of Technology as well as the Research Ethics Committee of Hiroshima University (permission nr. 172). Written consent was obtained from all subjects participating in the study (approved by the Research Ethics Committee of the Okinawa Institute of Science and Technology and the Research Ethics Committee of Hiroshima University).

The objective here is to explore ways of analyzing the results from our multiple co-clustering method in a real situation where the true subject-cluster structures are unknown. The depression data comprise 125 subjects (66 healthy and 59 depressive) and 243 features (S1 Table) that were collected at a collaborating university. Among these features, there are 129 numerical (e.g., age, severity scores of psychiatric disorders) and 114 categorical features (e.g., sex, genotype) with a number of missing entries. Importantly, these data were collected from subjects in three different phases. The first phase was when depressive subjects visited a hospital for the first time. The second phase was 6 weeks after subjects started medical treatment. The third phase was 6 months after the onset of the treatment. For healthy subjects, relevant data for the second and the third phases were not available (dealt as missing entries in the data matrix). To distinguish between these phase differences, we denote features in the second and the third phases with endings of 6*w* and 6*m*, respectively. Further, we did not include the label of health/depression status for clustering. We used it only for interpretation of results. We assumed that numerical features follow mixtures of Gaussian distributions in our model. To pre-process numerical features, we standardized each of them using means and standard deviations of available (i.e., non-missing) entries.

#### Results

**Fig 12:**
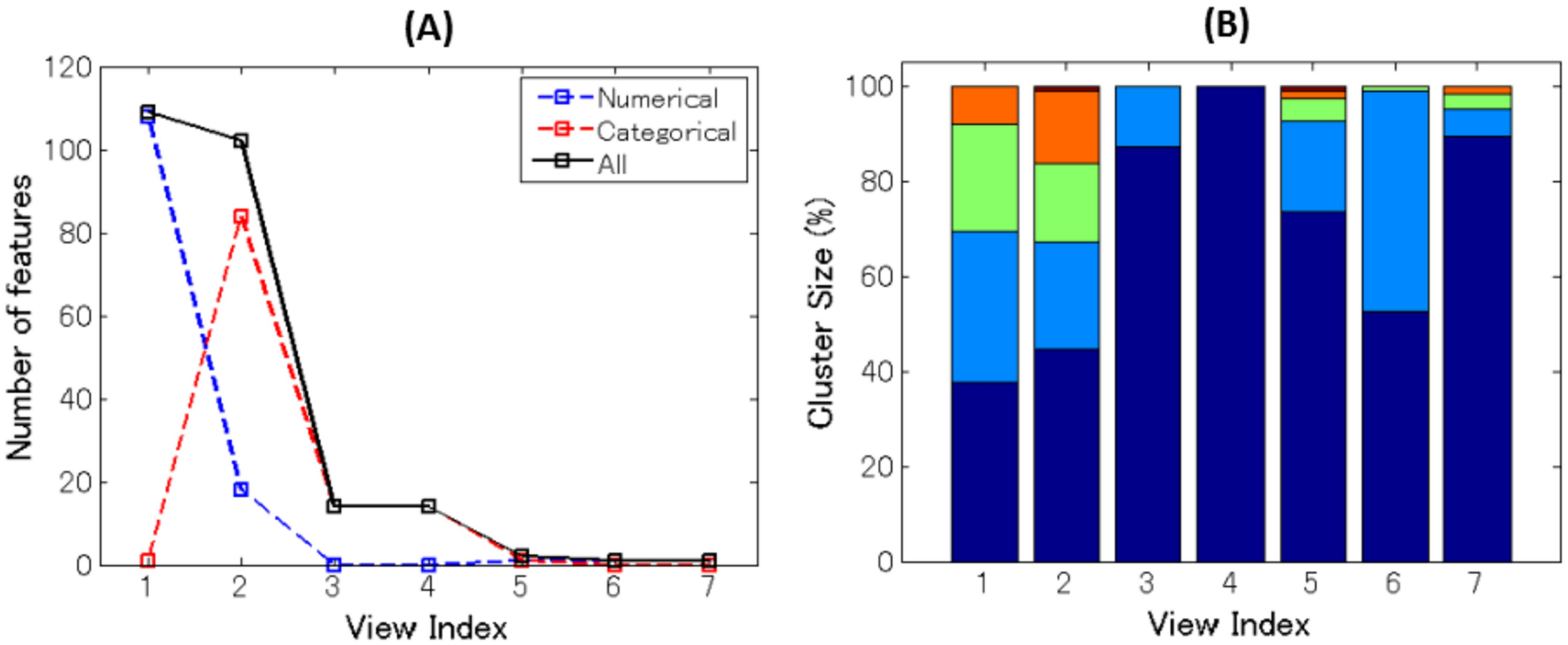
Results of the multiple co-clustering method for clinical data of depression. Panel (A): Number of features (in black) in each view with numerical features in blue and categorical features in red. Panel (B): cluster size (percentage of subjects) for subject clusters in each view.

**Fig 13:**
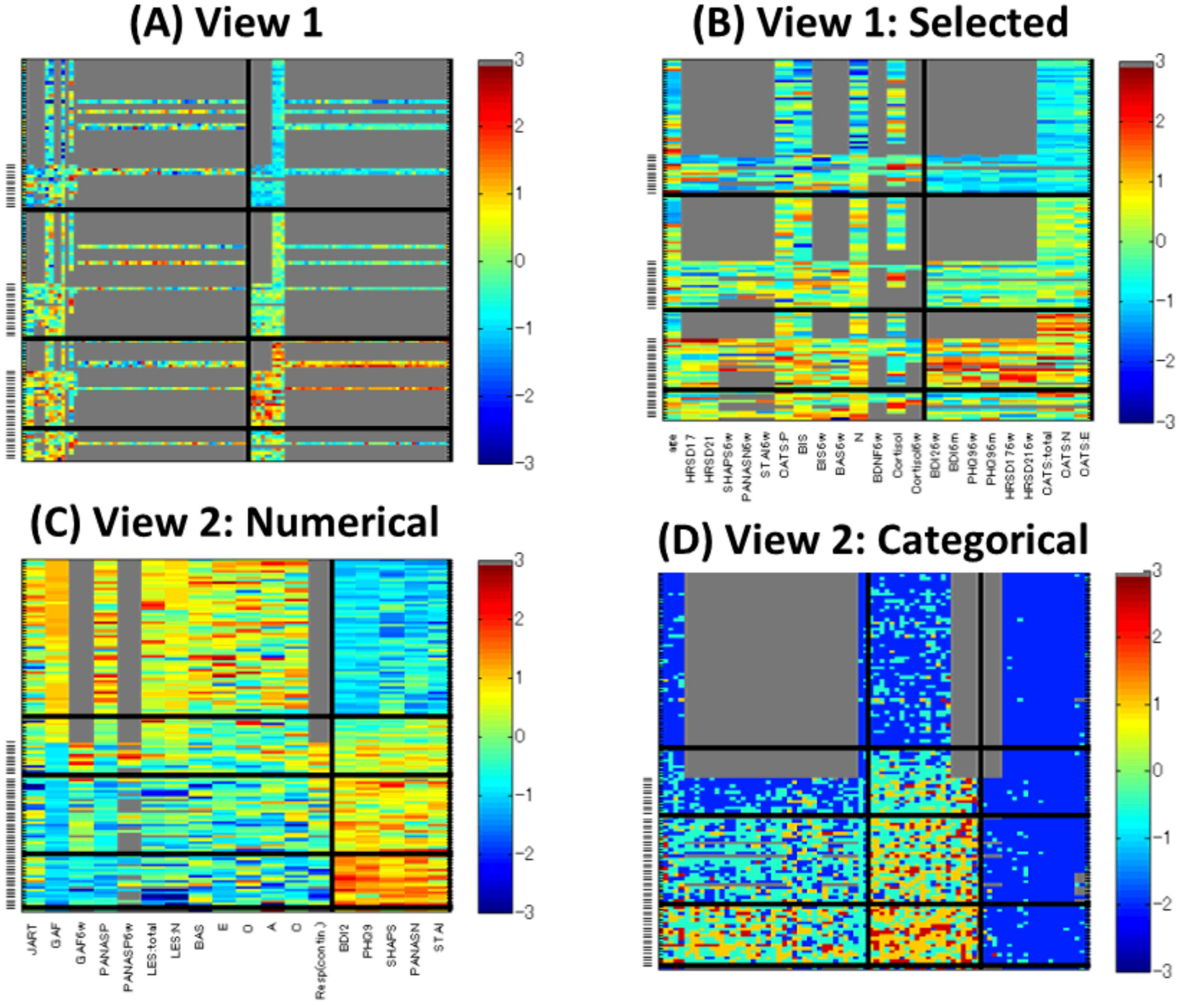
Visualizations of views yielded by our multiple co-clustering method. Panels (A)-(B): Heatmaps of views 1. The x-axis denotes numerical features, and the y-axis denotes subjects. A depressive subject is indicated by a hyphen in left. The subject clusters are sorted in the order of cluster size. Panel (B) is a copy of panel (A) after removing methylation related features (those having a large number of missing entries). Panels (C)-(D): Heatmaps of views 2. Panel (C) contains numerical features while panel (D) contains categorical ones. The subject clusters are sorted in the descending order of the proportion of depressive subjects. For these panels, the subjects within a subject cluster are sorted in the order of healthy and depressive subjects. On the other hand, feature clusters are sorted in the order of feature clusters in the order of feature cluster size. Note that for categorical features the color is arbitrary and that missing entries are in gray.

**Fig 14:**
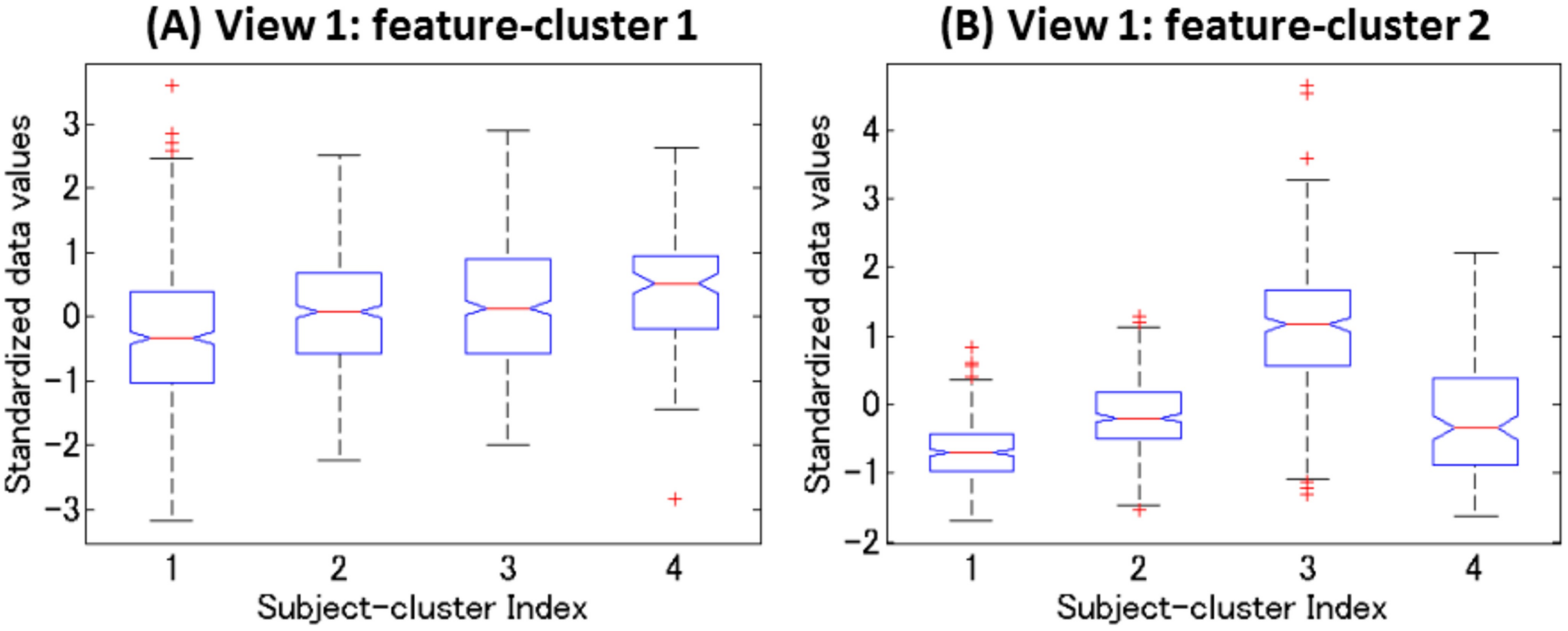
Distributions of the standardized data. Panel (A) for feature cluster 1 Panel (B) for feature cluster 2. X-axis denotes subject cluster index. All relevant entries except for missing ones are accommodated in each box.

Our multiple co-clustering method yielded seven views. The majority of features are allocated to two views (view 1 and view 2 in Fig 12A). The number of subject clusters ranges from one to five (Fig 12B). We analyze these cluster results more in detail, focusing on view 1 and view 2. View 1 has two feature clusters for numerical features, in which the majority of features are related to DNA methylation of CpG sites of the trkb and htr2c genes with a number of missing entries (Fig 13A). For better visualization of this view, we remove methylation-related features (Fig 13B). Among these two (numerical) feature clusters, feature cluster 1 does not discriminate well between the yielded subject clusters (Fig 14A), while feature cluster 2 does well (Fig 14B). Hence, subject clustering in this views is largely characterized by features in feature cluster 2 (BDI26w, BDI26m, PHQ96w, PHQ96m, HRSD176w, HRSD216w, CATS:total, CATS:N, and CATS:E). The first six features are related to psychiatric disorder scores at six weeks (features ending with -6*w*) and six months (features ending with -6*m*) after the onset of depression treatment. Hence, we can interpret this to reflect treatment effects. On the other hand, CATS:total, CATS:N, and CATS:E are related to abusive experiences in the subject’s childhood. Hence, these features are available before the onset of treatment. These data attributes suggest that it is possible to predict treatment effect by using features related to child abuse experiences. In particular, the distribution pattern in subject cluster 3 (Fig 13B) is remarkably different from those in the remaining subject clusters (Fig 14B).

In view 2, healthy and depressive subjects are well separated. The first subject cluster is for healthy subjects. The second is intermediate, and the third and fourth are for depressive subjects (Fig 13C). Relevant numerical features are: JART, GAF, GAF6w, PANASP, PANASP6w, LES:total, LES:N, BAS, E, O, A, C, and Rep. in feature cluster 1 and BDI2, PHQ9, SHAPS, PANASN, and STAI in feature cluster 2. This result is quite reasonable, because the majority of these features are scores from clinical questionnaires that evaluate depressive disorders either negatively (smaller values in feature cluster 1) or positively (larger values in feature cluster 2). The result for categorical features is displayed in Fig 13D. We do not go into detail analysis, but it is observed that clear differences of distributions between subject clusters are observed in feature cluster 1 and 2.

### Comparison of time complexity

Finally, we briefly discuss complexity of the clustering methods. Except for COALA, we need to run clustering methods (i.e., multiple co-clustering, decorrelated *K*-means, and restricted multiple clustering) with a number of random initializations for their parameters, and choose the optimal solution. Hence, computation time depends on the number of initializations. To compare complexity of computation, we make several assumptions. First, we focus on a single run of each method. Second, we assume the same number of iterations for convergence. Third, we assume that the numbers of views and clusters are fixed. In such a setting, time complexity for our multiple clustering method (as well as the restricted multiple clustering) is *O*(*nd*), where *n* and *d* are the number of samples and the number of features, respectively. This suggests that the complexity is just linear when either *n* or *d* is fixed. On the other hand, the complexities of COALA and decorrelated *K*-means are *O*(*n*^2^ log *n* + *n*^2^ *d*) and *O*(*nd* + *d*^3^), respectively, based on their typical algorithms [13, 16]. These results imply that the complexity of our multiple co-clustering is generally less than those of COALA and decorrelated *K*-means, suggesting superior efficiency of the present method. Indeed, in the simulation of facial image data, our multiple clustering method requires less time per run than COALA and decorrelated *K*-means (Fig 8C).

## Discussion

We proposed a novel method of multiple clustering in which each view comprises a co-clustering structure, and each cluster block fits a (heterogeneous) univariate distribution. Though our method assumes a somewhat complicated cluster structure (multiple views of co-clustering structures), it effectively detects multiple cluster solutions by clustering relevant features within a view, based on their distributional patterns. In contrast with our multiple co-clustering method, the restricted multiple clustering method is simple and straightforward for implementation. However, from a factor-analytical perspective, fitting a single distribution to all features in a view implies the dimensional reduction of that view by a single factor, which may be too restrictive for high-dimensional data. On the other hand, our method relaxes this constraint, allowing possible factors to be inferred in a data-driven approach. Practically, if there is prior knowledge that each view consists of a single factor, then we may use the restricted multiple clustering method. Otherwise, it is preferable to use our multiple co-clustering method, as demonstrated in both synthetic and real data applications above.

In comparisons with COALA and decorrelated *K*-means methods, our multiple co-clustering method outperforms other state-of-the-art methods using facial image and cardiac arrhythmia data. Beyond its better performance in sample clustering, our multiple co-clustering method has several advantages over other methods. It can infer the number of views/clusters. It is applicable to datasets comprising different types of features, and it can identify relevant features. Furthermore, our method is computationally efficient. The reason for this efficiency is the fitting of a univariate distribution to each cluster block. It is notable that despite using only a univariate distribution, our multiple co-clustering method can flexibly fit a dataset by adapting the number of views/clusters by means of a Dirichlet process.

It is also worth noting that the multiple co-clustering method is not only useful to recover multiple cluster structures of data, but also a single-cluster structure. In the case of single clustering, our method works by selecting relevant features for possible sample clustering. This may be the main reason that our method performs better with the cardiac arrhythmia data than COALA and decorrelated *K* means, which use the data without feature selection.

Finally, we discuss limitations of the proposed method. First, the optimization algorithm laid out in Algorithm 1 may not be efficient for a dataset of large size. The optimization algorithm ensures only local optimization: Lower bound L of likelihood in Eq (3) monotonously increases as the hyperparameters are updated. However, it does not guarantee the global optimization. Hence, to obtain a better solution, we need a large number of runs of the algorithm with different initializations. To improve efficiency of searching the global optimized solution, it will be useful to perform parallel computation with different initializations, and/or to combine our algorithm with stochastic search algorithm, which widens the scope of search space without being trapped in a local model. Second, our method does not capture relationships among feature clusters. Hence, negatively correlated features are allocated to different feature clusters, which requires careful interpretations of feature clusters. It is recommended to visualize feature cluster solutions or evaluating correlation coefficients among features in different feature clusters. Third, as has been illustrated in the simulation study on a large number of views, our method may not be able to detect a large number views even when these views are clearly distinguishable. Nonetheless, the simulation study suggested that different views merged into a single view. Hence, it is recommended to re-run our method for a particular view if the object-cluster structure in the view does not clearly show up.

## Supporting information

S1 Appendix Observation models for Gaussian, Poisson and multinomial. Observation models for Gaussian, Poisson and multinomial distributions with priors, the expectation of log-likelihood and the updating equations.
(PDF)

S1 Table List of features for clinical data. List of all features used in Depression Data.
(PDF)
