# Supplementary material for: Multiple co-clustering based on nonparametric mixture models with heterogeneous marginal distributions
Source: PLoS One. 2017 Oct 19;12(10):e0186566. doi: 10.1371/journal.pone.0186566 (PMC5648298; doi:10.1371/journal.pone.0186566)
Supplement: S1 Appendix — Observation models for Gaussian, Poisson and multinomial distributions with priors, the expectation of log-likelihood and the updating equations. (PDF) [file pone.0186566.s001.pdf]

S1 Appendix: Observation models for Gaussian, Poisson and multinomial

This is a supplementary material for observation models in the main manuscript, providing priors, the expectation of log-likelihood and the updating equations.

Gaussian distribution

We denote univariate Gaussian density function as $\mathrm{Gauss}(\cdot|\mu, \sigma^2)$ where μ and σ^2 are mean and variances. We assume conjugate priors for μ and σ^2 in each cluster block:

$$\begin{aligned} s_{v,g,k} &\sim \mathrm{Ga}(\cdot|\gamma_0/2, \gamma_0\sigma_0^2/2) \\ \mu_{v,g,k} &\sim \mathrm{Gauss}(\cdot|\mu_0, (\lambda_0 s_{v,g,k})^{-1}), \end{aligned}$$

where $\mathrm{Ga}(\cdot|a, b)$ denotes Gamma distribution with shape and rate parameters (a, b). In the present paper, we set $\sigma_0^2 = 10^4$, $\gamma_0 = 1$, and $\lambda_0 = 10^{-4}$ so that the prior distributions are nearly non-informative. It can be shown that the variational approximation for the posterior $q_{\boldsymbol{\theta}^{(m)}}(\boldsymbol{\theta}^{(m)})$ is given by

$$\prod_{v=1}^{V}\prod_{g=1}^{G}\prod_{k=1}^{K} \mathrm{Gauss}(\mu_{v,g,k}|\mu_{0,v,g,k}, (\lambda_{0,v,g,k} s_{0,v,g,k})^{-1}) \\ \times \mathrm{Ga}(s_{0,v,g,k}|\gamma_{0,v,g,k}/2, \gamma_{0,v,g,k}\sigma_{0,v,g,k}^2/2),$$

where the hyperparameters are updated by

$$
\begin{aligned}
\lambda_{0,v,g,k} &= \lambda_0 + \sum_{j=1}^{d^{(m)}} \sum_{i=1}^{n} \tau_{j,v,g}^{(m)} \eta_{i,v,k} \\
\mu_{0,v,g,k} &= \frac{1}{\lambda_{0,v,g,k}} \Big\{ \lambda_0 \mu_0 + \sum_{j=1}^{d^{(m)}} \sum_{i=1}^{n} \tau_{j,v,g}^{(m)} \eta_{i,v,k} X_{i,j}^{(m)} \Big\} \\
\gamma_{0,v,g,k} &= \gamma_0 + \sum_{j=1}^{d^{(m)}} \sum_{i=1}^{n} \tau_{j,v,g}^{(m)} \eta_{i,v,k} \\
\sigma_{0,v,g,k}^2 &= \frac{1}{\gamma_{0,v,g,k}} \Big\{ \gamma_0 \sigma_0^2 + \lambda_0 \mu_0^2 \\
&+ \sum_{j=1}^{d^{(m)}} \sum_{i=1}^{n} \tau_{j,v,g}^{(m)} \eta_{i,v,k} (X_{i,j}^{(m)})^2 - \lambda_{0,v,g,k} \mu_{0,v,g,k}^2 \Big\}.
\end{aligned}
$$

Finally, the expectation of the conditional log-likelihood $\mathbb{E}_{q(\boldsymbol{\theta})}\Big[\log p(X_{i,j}^{(m)} | \boldsymbol{\theta}_{v,g,k}^{(m)}) \Big]$ is given by

$$
\begin{aligned}
-\frac{1}{2} \Big\{ & \frac{(X_{i,j}^{(m)} - \mu_{0,v,g,k})^2}{\sigma_{0,v,g,k}^2} + \frac{1}{\lambda_{0,v,g,k}} + \log \sigma_{0,v,g,k}^2 \\
& + \log(\gamma_{0,v,g,k}/2) - \psi(\gamma_{0,v,g,k}/2) + \log(2\pi) \Big\}.
\end{aligned}
$$

Poisson distribution

We denote Poisson distribution as $\text{Poisson}(\cdot|\lambda)$ where λ is a rate parameter. The conjugate prior for λ is given by

$$
\lambda_{v,g,k} \sim \text{Ga}(\cdot|\alpha_0, \beta_0),
$$

where we set α_0 and β_0 to one. It can be shown that the variational approximation is given by

$$
q_{\boldsymbol{\theta}^{(m)}}(\boldsymbol{\theta}^{(m)}) = \prod_{v=1}^{V} \prod_{g=1}^{G} \prod_{k=1}^{K} \text{Ga}(\lambda_{v,g,k} | \alpha_{0,v,g,k}, \beta_{0,v,g,k}),
$$

where the hyperparameters are updated by

$$\begin{aligned}\alpha_{0,v,g,k} &= \alpha_0 + \sum_{j=1}^{d^{(m)}} \sum_{i=1}^{n} \tau_{j,v,g}^{(m)} \eta_{i,v,k} X_{i,j}^{(m)} \\ \beta_{0,v,g,k} &= \beta_0 + \sum_{j=1}^{d^{(m)}} \sum_{i=1}^{n} \tau_{j,v,g}^{(m)} \eta_{i,v,k}.\end{aligned}$$

The expectation of the conditional log-likelihood becomes

$$X_{i,j}^{(m)} \{\psi(\alpha_{0,v,g,k}) - \psi(\beta_{0,v,g,k})\} \\ - \frac{\alpha_{0,v,g,k}}{\beta_{0,v,g,k}} - \sum_{t=1}^{X_{i,j}^{(m)}} \log t.$$

Categorical/multinomial distribution

For a categorical feature x ($x \in \{c_1, \ldots, c_H\}$), we denote categorical distribution as $\mathrm{Cat}(\cdot|\boldsymbol{p})$ where H is the number of categories, and $\boldsymbol{p} = (p_1, \ldots, p_H)$ are probabilities for each category with $\sum_{h=1}^{H} p_h = 1$. We assume the conjugate prior for $(p_1, \ldots, p_H)$,

$$(p_1, \ldots, p_H) \sim \mathrm{Dirichlet}(\cdot|\boldsymbol{\rho}_0),$$

where $\mathrm{Dirichlet}(\cdot|\boldsymbol{\rho}_0)$ denotes a Dirichlet distribution with prior sample size $\boldsymbol{\rho}_0$. We set $\boldsymbol{\rho}_0$ to $(1, \ldots, 1)$. It can be shown that

$$q_{\boldsymbol{\theta}^{(m)}}(\boldsymbol{\theta}^{(m)}) = \prod_{v=1}^{V} \prod_{g=1}^{G} \prod_{k=1}^{K} \mathrm{Dirichlet}(\boldsymbol{p}_{v,g,k}|\boldsymbol{\rho}_{0,v,g,k}),$$

where the hyperparameters are updated by

$$\rho_{0,v,g,k,h} = \rho_{0,h} + \sum_{j=1}^{d^{(m)}} \sum_{i=1}^{n} \tau_{j,v,g}^{(m)} \eta_{i,v,k} \mathbb{I}(X_{i,j}^{(m)} = c_h),$$

where $\rho_{0,v,g,k,h}$ denotes the hth element of $\boldsymbol{\rho}_{0,v,g,k}$. The expectation of the log-likelihood is then given by

$$\sum_{h=1}^{H} \mathbb{I}(X_{i,j}^{(m)} = c_h)\{\psi(\rho_{0,h,v,g,k}) - \psi(\sum_{h'=1}^{H} \rho_{0,h',v,g,k})\}.$$

Since the categorical distribution differs depending on the number of categories H, we need to define different types of categorical distribution. Alternatively, for the purpose of simplicity, we can set H to the maximum number of categories for different categorical features, and fit a single family of categorical distribution to all these features.

More generally, in the case of multinomial distribution, the update equation and the expectation of the log-likelihood becomes

$$\rho_{0,v,g,k,h} = \rho_{0,h} + \sum_{j=1}^{d^{(m)}} \sum_{i=1}^{n} \tau^{(m)} \eta_{i,v,k} n_{i,j,h}$$

$$\sum_{h=1}^{H} n_{i,j,h} \{\psi(\rho_{0,h,v,g,k}) - \psi(\sum_{h'=1}^{H} \rho_{0,h',v,g,k})\} + \log \binom{\sum_{h=1}^{H} n_{i,j,h}}{n_{i,j,1}, \ldots, n_{i,j,H}},$$

where $n_{i,j,h}$ is the number of category c_h in $X_{i,j}^{(m)}$; the last term is the logarithm of multinomial coefficients.
