# Supplementary material for: Multiple co-clustering based on nonparametric mixture models with heterogeneous marginal distributions
Source: PLoS One. 2017 Oct 19;12(10):e0186566. doi: 10.1371/journal.pone.0186566 (PMC5648298; doi:10.1371/journal.pone.0186566)
Supplement: S1 Table — List of all features used in Depression Data. (PDF) [file pone.0186566.s002.pdf]

S1 Table: List of features for clinical data

Features
**Numerical features**
BAS (Behavioral Activation Scale),
BDNF (Quantity of brain-derived neurotrophic factor in blood),
BDI2 (Beck Depression Inventory),
BIS (Behavioral Inhibition Scale),
CATS (Child Abuse and Trauma Scale),
Cortisol (Quantity of cortisol in blood),
GAF (Global Assessment of Functioning),
PHQ9 (Patient Health Questionnaire),
HRSD17 (Hamilton Rating Scale for Depression),
JART (Adult reading test),
LES (Life Experiences Survey),
PANASP (Positive Affect Schedule),
PANASN (Negative Affect Schedule),
SHAPS (Snaith-Hamilton Pleasure Scale),
STAI (State-Trait Anxiety Inventory),
N, E, O, A, C<br>(Five factors in revised NEO Personality Inventory)
**Categorical features**
Sex
miniA-P (Mini-International Neuropsychiatric Interview),<br>*A-P corresponds to the following psychiatric symptoms*:<br>Major depressive disorder (A),<br>Dysthymia (B), Suicide risk (C),<br>Mania (D), Panic disorder (E), Agoraphobia (F),<br>Social phobia (G),<br>Obsessive compulsive disorder (H),<br>PTSD (I), Alcohol dependence and abuse (J),<br>Drug dependence and abuse (K),<br>Psychotic disorder (L), Anorexia (M), Bulimia (N),<br>Generalized anxiety disorder (O),<br>Antisocial personality disorder (P),
SNPs 1-8: Single Nucleotide Polymorphisms that are located in the following genome sites, respectively. (in parenthesis are the relevant gene functions)<br>rs1187323 (NTRK2), rs34118353 (5HT1a receptor),<br>rs3756318 (NTRK2), rs3813929 (5HT2c receptor),<br>rs45554739 (NTRK2), rs56384968 (SLC6A4),<br>rs6265 (BDNF), rs6294 (5HT1a receptor)
